# *Rad21*-Cohesin Haploinsufficiency Impedes DNA Repair and Enhances Gastrointestinal Radiosensitivity in Mice

**DOI:** 10.1371/journal.pone.0012112

**Published:** 2010-08-12

**Authors:** Huiling Xu, Kuhendra Balakrishnan, Jordane Malaterre, Matthew Beasley, Yuqian Yan, Jeroen Essers, Esther Appeldoorn, Jonathan M. Thomaszewski, Melisa Vazquez, Sandra Verschoor, Martin F. Lavin, Ivan Bertonchello, Robert G. Ramsay, Michael J. McKay

**Affiliations:** 1 Research Division, Peter MacCallum Cancer Centre, Melbourne, Victoria, Australia; 2 Department of Pathology, Faculty of Medicine and Dental Sciences, The University of Melbourne, Parkville, Victoria, Australia; 3 Department of Cell Biology and Genetics, Erasmus Medical Centre, Rotterdam, The Netherlands; 4 Department of Cell Biology and Genetics, Department of Radiobiology, Department of Vascular Surgery, Erasmus Medical Centre, Rotterdam, The Netherlands; 5 Division of Radiation Oncology, Peter MacCallum Cancer Centre, Melbourne, Victoria, Australia; 6 Radiation Biology and Oncology, Queensland Institute of Medical Research, Queensland, Australia; 7 Department of Pharmacology, The University of Melbourne, Parkville, Victoria, Australia; University Medical Center Hamburg-Eppendorf, Germany

## Abstract

Approximately half of cancer-affected patients receive radiotherapy (RT). The doses delivered have been determined upon empirical experience based upon average radiation responses. Ideally higher curative radiation doses might be employed in patients with genuinely normal radiation responses and importantly radiation hypersensitive patients would be spared the consequences of excessive tissue damage if they were indentified before treatment. *Rad21* is an integral subunit of the cohesin complex, which regulates chromosome segregation and DNA damage responses in eukaryotes. We show here, by targeted inactivation of this key cohesin component in mice, that *Rad21* is a DNA-damage response gene that markedly affects animal and cell survival. Biallelic deletion of *Rad21* results in early embryonic death. *Rad21* heterozygous mutant cells are defective in homologous recombination (HR)-mediated gene targeting and sister chromatid exchanges. *Rad21^+/−^* animals exhibited sensitivity considerably greater than control littermates when challenged with whole body irradiation (WBI). Importantly, *Rad21^+/−^* animals are significantly more sensitive to WBI than *Atm* heterozygous mutant mice. Since supralethal WBI of mammals most typically leads to death *via* damage to the gastrointestinal tract (GIT) or the haematopoietic system, we determined the functional status of these organs in the irradiated animals. We found evidence for GIT hypersensitivity of the *Rad21* mutants and impaired bone marrow stem cell clonogenic regeneration. These data indicate that *Rad21* gene dosage is critical for the ionising radiation (IR) response. *Rad21* mutant mice thus represent a new mammalian model for understanding the molecular basis of irradiation effects on normal tissues and have important implications in the understanding of acute radiation toxicity in normal tissues.

## Introduction

Radiotherapy (RT) is employed in approximately half of all cancer patients. Most RT is delivered to a cancer-affected organ, unavoidably encapsulating normal tissues surrounding a tumor. Optimal outcomes are a balance between normal tissue toxicity and tumour control: this balance is known as the therapeutic ratio. In some cases, RT is delivered with very large fields, such as for total/whole body irradiation (T/WBI), which is used in hematopoietic stem cell transplantation in patients with hematologic and other malignancies.

Although some rare syndromes are characterised by radiosensitivity, mechanistic insights of the effects of IR on the majority of patients' normal tissues and tumours are largely lacking. For example rare (1 in 100,000–200,000 newborns) *Ataxia telangiectasia* (*Atm*) patients are hypersensitive to RT [1]–[4]. While *Atm* heterozygous carriers are relatively common (1 in 200 individuals) and have an elevated risk for cancer particularly breast cancer [5]–[7], they are not reliably identified by hypersensitivity to RT or to IR *in vitro*. Identification of potential new mammalian radiation responsive genes/pathways promises refinement of treatment protocols and the possibility of tailored therapy. Nevertheless this has been an elusive goal.

*Rad21* (also known as *Scc1 or MCD1*) was initially identified in a genetic screen in fission yeast (*Saccharomyces pombe)* [8]. This clone contains a point mutation in one allele of the *Rad21* gene and exhibited hypersensitivity to radiation owing to its impaired double strand DNA breakage repair [8]. Recent studies in lower eukaryotes and metazoa have shown that paralog mammalian *Rad21* genes are key regulatory components of a multi-protein complex, cohesin [9]–[14]. Cohesin plays an essential role in mediating sister chromatid cohesion (SCC), a mechanism critical for proper chromosome segregation [10]–[12]. Biallelic deletion of cohesin subunits results in cell death [10]–[12]. Recent studies implicate cohesin in the DNA damage response and repair in eukaryotic cells [13]–[16]. We previously found *Rad21* variants in cancer patients exhibiting acute radiation toxicity, suggesting an association between *Rad21* gene variants and normal tissue protection that may be defective in some radiation sensitive cancer patients [17].

To gain insight into the contribution of *Rad21* cohesin to normal tissue IR toxicity, we generated mutant mice with one functional allele of *Rad21* and investigated the IR response of this mutant in the context of whole animals. Our study provides the first evidence that *Rad21,* and possibly cohesin and their associated genes, represent a new class of novel total body radiation response gene(s) in mammals, the characterisation of which has important implications for patient-tailored cancer therapy and modulating normal tissue responses to clinical RT.

## Results

### Deletion of the mouse *Rad21* gene results in early embryonic lethality

To generate a null allele of the mouse *Rad21* gene, we constructed a targeting vector which replaced exon 2 of the mouse *Rad21* gene with a *neo* resistance cassette flanked by *lox^P^* sites, resulting in a null allele (Fig. S1A). The targeting construct was introduced to mouse embryonic stem (ES) cells and mutant cells were produced by homologous recombination. Targeted alleles were identified by PCR and Southern blot analysis (Fig. S1A).

Chimeric offspring were obtained from *Rad21^+/−^* ES cell clone 5 which showed a reduced RAD21 protein level (Fig. S1B) and heterozygous *Rad21^+/−^* mice were obtained by breeding. *Rad21^+/−^*mice were viable and developed to apparently normal adulthood without morphological defects. Genotyping of 255 live-born offspring (n = 40 litters) from heterozygous parents revealed no homozygous animals, suggesting that homozygous null *Rad21* mice die *in utero*. Further examination of embryos at embryonic gestational times of E8.5 (n = 7), E10.5 (n = 15) and E13.5 (n = 25) days from heterozygous parents, revealed no homozygous mutants at all three developmental stages examined. Together, these data indicate that homozygous *Rad21* deficiency leads to early embryonic lethality. Thus, at least one WT *Rad21* allele is essential for normal development in mammals and the generation of homozygous knock-out cells and embryos for study was precluded by this traditional approach. Nevertheless, *Rad21* heterozygous cells and mice were available for investigation.

### *Rad21^+/−^* mouse embryonic fibroblasts (MEFs) exhibit increased chromosomal number abnormality and mitotic defects

Deletion of *Rad21* compromise proper chromosome segregation, leading to gains or losses of chromosomes (aneuploidy) in yeast and vertebrates [10], [11], [14]. To determine whether chromosomal number is altered in heterozygous *Rad21* knock-out cells, we examined metaphase chromosome spreads in early passage MEFs from *Rad21^+/−^* and isogenic WT mice. Cells with diploid chromosome numbers (*i.e.* 40 chromosomes) were observed in approximately 56% of WT cells (Fig. S2A). In contrast, a clear reduction in cells with diploid chromosome content was observed in *Rad21^+/−^* MEFs (Fig. S2A). This was accompanied by an increase in the percentage of aneuploid cells (Fig. S2A). Further examination of chromosome spreads showed that *Rad21^+/−^* cells often had one or more pairs of sister chromatids with no apparent connection at centromeres, suggesting the premature loss of, or weakened centromeric cohesion, or an altered centric chromatin organization (Fig. S2B). Furthermore, *Rad21^+/−^* MEFs showed more frequent mitotic defects compared to WT (Fig. S2C). The commonly observed mitotic defects in *Rad21^+/−^* MEFs include uncondensed or mis-aligned chromosomes at metaphase, chromosome bridges and lagging at anaphase, and multipolar mitosis (Fig. S2C).

### Response of *Rad21^+/−^* cells to DNA damaging agents

**Figure 1:**
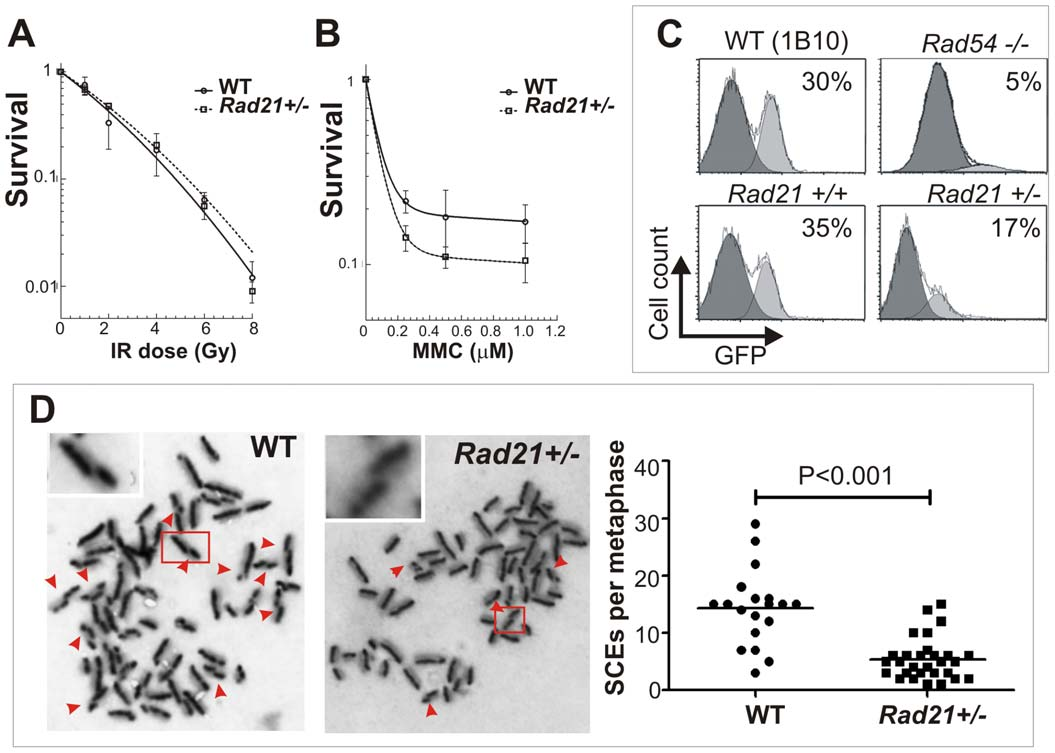
HR deficiency of *Rad21^+/−^* cells. **A**. Clonogenic survival of WT and isogenic *Rad21^+/−^* ES cells following IR. **B**. Clonogenic survival of *Rad21^+/−^* ES cells following MMC indicated that *Rad21^+/−^* ES cells were more sensitive to MMC than WT ES cells. **C**. Gene targeting efficiency in *Rad21^+/−^* ES cells. *Rad54^−/−^* and its isogenic parental 1B10 ES cell line were used as gene targeting-defective and -proficient controls, respectively. **D**. SCE frequency in WT and isogenic *Rad21^+/−^* MEFs following treatment with 6 µM MMC. Red arrowheads indicate SCEs. *Inserts: enlarged images of boxed regions.* The number of metaphase spreads scored: WT n = 19; *Rad21^+/−^* n = 29. Horizontal bars represent the respective means.

Both budding and fission yeast *Rad21* mutants are hypersensitive to IR and defective in DNA repair[8], [18]. Likewise, a conditional *Rad21* deletion in chicken DT-40 cells led to an increase in spontaneous and IR-induced chromatid breaks [14]. To test whether *Rad21^+/−^* mouse cells are IR sensitive, we determined the clonogenic survivals of WT and *Rad21^+/−^* ES cells following IR. No significant difference was observed between the *Rad21^+/−^* and WT cells at any dose tested (Fig. 1A). We then examined the clonogenic survival of *Rad21^+/−^* ES cells after treatment with the DNA cross-linking agent, Mitomycin C (MMC) in view of the evidence that the predominant means of DNA repair of MMC-induced damage is mediated by HR repair [19]. In response to MMC treatment, *Rad21^+/−^* ES cells displayed a significantly reduced survival compared to WT (p<0.001) (Fig. 1B).

### Reduced gene targeting efficiency at an independent locus, and impaired MMC-induced SCE formation in *Rad21^+/−^* cells reveal *in vivo* and *in vitro* HR deficiency

Hypersensitivity of *Rad21^+/−^* cells to cross-linking agents like MMC raises the prospects of an associated defect in HR repair pathways. Certainly, SCC genes have been implicated in promoting HR [13], [16]. However, it is unknown if the HR role of SCC genes is conserved in mammalian cells. We therefore assessed the HR capability of *Rad21* ***^+/−^*** cells using an eGFP-based assay which employs a *Rad54*-GFP knock-in construct targeting the genomic *Rad54* locus in ES cells [20]. In this gene targeting assay, cells are transfected with the promoter-less hRad54GFP-puro knock-in targeting construct. Cells containing integrated construct (*via* gene targeting or random integration) were then selected in medium containing Puromycin. GFP-positive cells arise when the targeting construct integrates in the mRad54 locus *via* homologous recombination but not random construct integration. The frequency of these events is detected by GFP-fluorescence of targeted ES cells by flow cytometry, an indirect measure of targeting efficiencies. A *Rad54^−/−^* ES cell line was also employed as a gene targeting-defective control. As expected in *Rad54^−/−^* ES cells, there was a significant reduction in GFP-positive cells (5%) when compared to 30% GFP-positive cells in the WT isogenic cell line, 1B10 (Fig. 1C). The *Rad21* ***^+/−^*** ES cell line exhibited an approximately two-fold reduction in targeting efficiency (17%) when compared to its WT parental ES cells (35%), indicating a HR defect in live *Rad21^+/−^* ES cells. This result is in contrast to a previous report which showed an increased gene targeting efficiency in human 293 cells following a transient knockdown of *Rad21* by siRNA [16]. The discrepancy may be due to a specific function of RAD21 in HR, since the previous study measured episomal recombination following the site-specific induction of a DSB by the I-SceI endonuclease [16]. This targeting measures different recombination events than the homologous recombination event we measured by targeted integration. It is also possible that the severe deficiency of *Rad21* by siRNA knockdown may shift the preference to a different sub-pathway of HR.

To further probe the evidence for a role of *Rad21* in HR, we examined in *Rad21* mutant cells whether somatic recombination activity is also altered, by measuring the level of sister chromatid changes (SCEs) using *Rad21^+/−^* MEF cell lines derived from *Rad21^+/−^* mice. SCE has been shown to be closely associated with HR in vertebrate cells and the frequency of SCE serves as a measure of recombination activity [21]. Both WT and *Rad21^+/−^* mutant MEFs showed only spontaneous SCE levels of three per metaphase spread (data not shown). No significant difference between WT and *Rad21^+/−^* MEFs was observed. However, following treatment with 6 µM MMC, *Rad21^+/−^* MEFs showed a modest increase in SCE frequency with an average of five SCEs per metaphase spread (Fig. 1D). In contrast, the frequency of SCEs in WT MEFs was elevated substantially to an average of fourteen SCEs per metaphase spread, significantly higher (p <0.0001) than that of *Rad21^+/−^* MEFs (Fig. 1D). The suppression of SCEs in *Rad21^+/−^* MEFs is similar to that observed in human cells following siRNA knockdown of *Rad21* [16] and in known recombination-defective cells [21], providing further evidence for defective HR in *Rad21^+/−^* MEFs.

### *Rad21^+/−^* MEFs are defective in the DNA damage checkpoint activation and DSB repair

**Figure 2:**
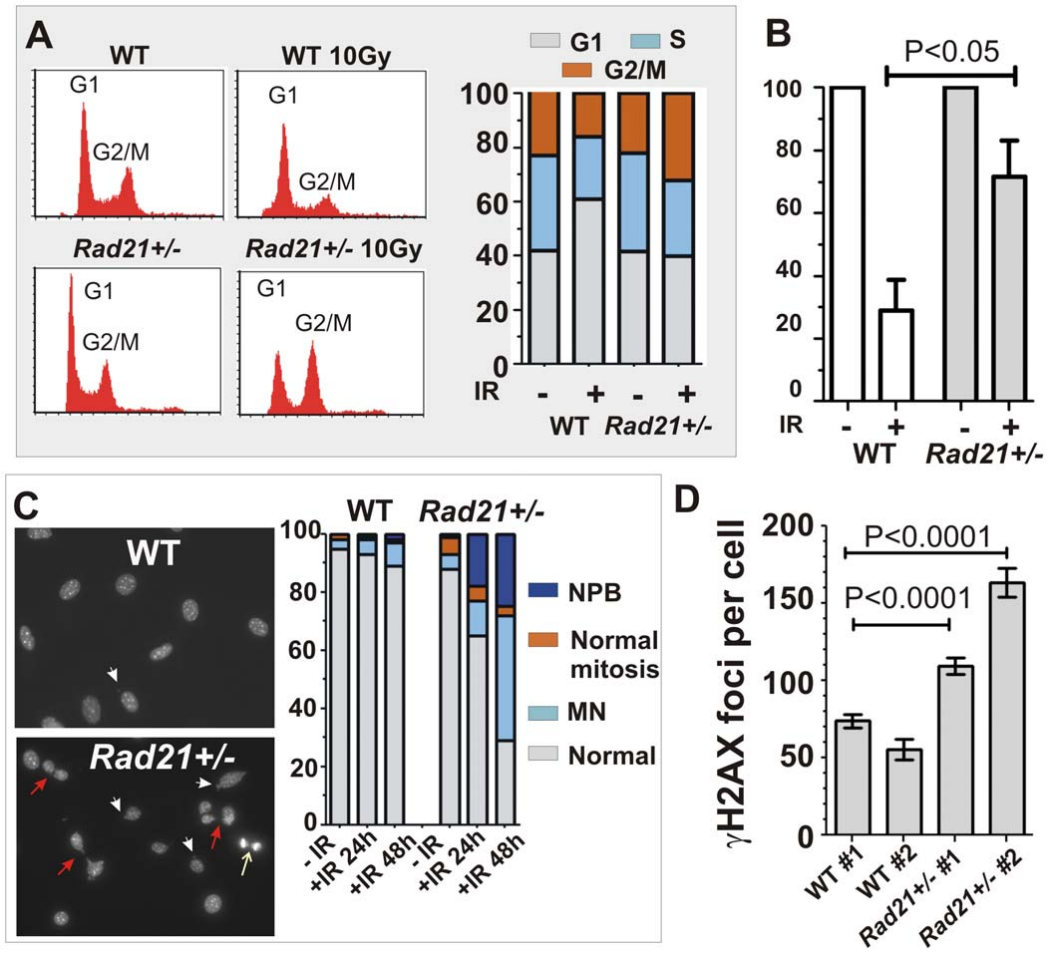
Radiation-induced DNA damage response of *Rad21^+/−^* cells. **A**. Cell cycle profiles of WT and isogenic *Rad21^+/−^* MEFs. Cells were analysed 24 hours post 10Gy IR, with unirradiated cells as controls. DNA content was used to determine the proportion of cells in different cell cycle phases. **B**. Change in the percentage of S-phase cells 24 hours post 10Gy IR. BrdU-positive cells were expressed as the percentage of unirradiated controls. Data represent means of three independently-derived MEF cell lines for each genotype. Error bars = SEM. **C**. Radiation-induced genomic instability. Nuclei were visualised by DAPI staining and scored at indicated time-points post 10Gy IR, for the presence of nucleoplasmic bridge (NPB, red arrows), micronuclei, MN (white arrowheads) and normal mitoses (white arrow). Also see Figure S4. The number of nuclei scored was as follows: WT unirradiated n = 643, 24 hours post IR n = 1131, 48 hours post IR n = 263; *Rad21^+/−^* unirradiated n = 782, 24 hours post IR n = 1298, 48 hours post IR n = 246. **D**. Quantification of radiation-induced γH2AX foci 4 hours post 10Gy IR. Two independently-derived WT and isogenic *Rad21^+/−^* MEF cell lines were used. The number of nuclei scored was as follows: WT #1 n = 172, WT #2 n = 303; *Rad21^+/−^* #1 n = 232, *Rad21^+/−^* #2 n = 104. Error bars = SEM.

Two key cellular responses to IR damage are checkpoint activation and DNA repair, the two processes being closely linked. To determine if *Rad21^+/−^* MEFs are defective in DNA damage-induced cell cycle arrest, we analysed cell-cycle profiles of *Rad21^+/−^* and isogenic WT MEF cells following IR. While similar cell cycle distributions were observed in unirradiated *Rad21^+/−^* and WT cells, the two cell types exhibited different profiles when examined 24 hours post IR (Fig. 2A). *Rad21^+/−^* cells showed a higher percentage of S phase cells compared to WT cells following IR (Fig. 2A). Further analysis using BrdU-pulse-labelling revealed approximately 71% reduction of cells undergoing DNA replication in WT MEFs following IR (Fig. 2B). Interestingly, there was only a modest reduction (approximately 29%) in BrdU-positive cells in *Rad21^+/−^* MEFs, following IR (Fig. 2B). These data showed that the majority of *Rad21^+/−^* cells continues to undergo DNA synthesis following IR, suggesting defective intra-S checkpoint activation. In addition to S phase cells, an increase in G2/M cells was observed in *Rad21^+/−^* MEFs following IR (Fig. 2A). Further examination of nuclear morphology using DAPI staining revealed that only 1% of mitotic cells were present in WT 24 hours post IR, indicating that WT cells were prevented from entering mitosis (Fig. 2C and Fig. S3). By contrast, 18% of *Rad21^+/−^* cells were engaged in mitosis. Strikingly, nucleoplasmic bridges (NPB) were present in most, if not all, mitotic *Rad21^+/−^* cells (Fig. 2C and Fig. S3), suggesting that a delayed progression through mitosis may contribute to NPB accumulation. Furthermore, cells with micronuclei (MN) were found to be more frequent in *Rad21^+/−^* MEFs (∼13%) than WT (5%). By 48 hours post IR, cells with NPB and MN were increased dramatically in *Rad21^+/−^* MEFs to 25% and 43%, respectively (Fig. 2C and Fig. S3). The presence of NPB and MN suggest that *Rad21^+/−^* cells more frequently enter mitosis with damaged DNA, resulting in both chromosomal and mitotic abnormalities.

We next examined whether IR-induced DSB repair is impaired in *Rad21^+/−^* cells using γH2AX as a reliable surrogate marker for DSBs [22]. Of two *Rad21^+/−^* MEF cell lines examined, both showed significantly higher numbers of γH2AX foci when compared to that of isogenic WT cells (Fig. 2D and Fig. S4). These data confirm that DSB repair is defective in *Rad21^+/−^* MEFs.

### *Rad21^+/−^* mice are sensitive to WBI

Although studies in *Rad21^+/−^* ES cells did not reveal apparent radiation hypersensitivity *in vitro,* we were mindful that *in vitro* survival assay may not be representative of somatic cell types *in vivo*. *Rad21^+/−^* mice are one of six thus far described cohesin mouse mutants [23]–[27], but to date these mutants have not been studied to assess the effect of cohesin deficiency on radiation response in the context of whole animals. This might be important with regard to patient responses to RT. Therefore we examined the effects of WBI on the survival of *Rad21^+/−^* mice.

**Figure 3:**
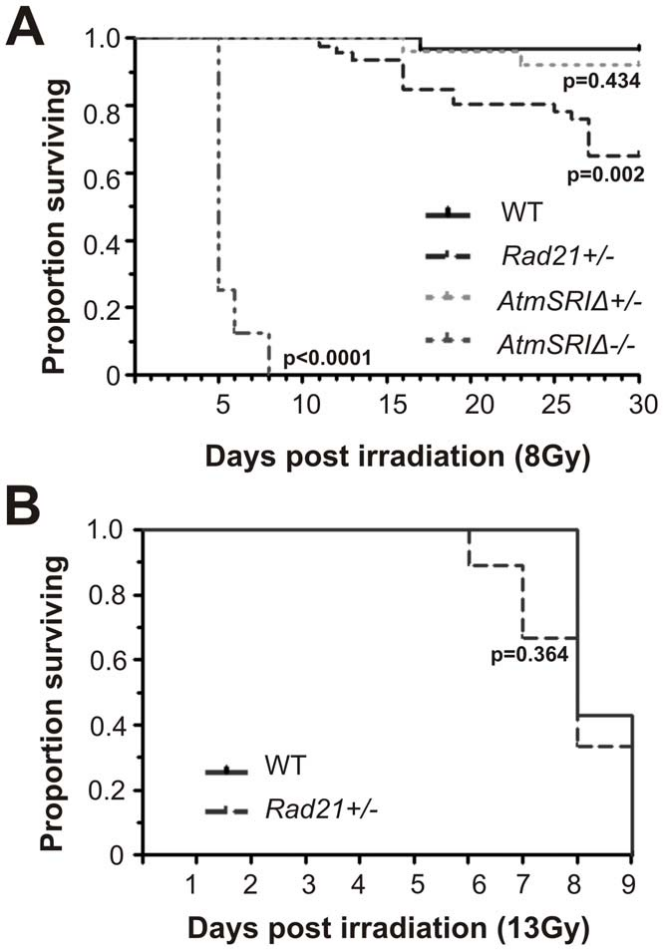
Kaplan-Meier plots of cumulative survival of *Rad21*-cohesin and *Atm* mutant animals following WBI. **A**. Cumulative survival following 8Gy WBI is shown. *Rad21* ^+/−^: n = 46; *AtmSRIΔ^+/−^* n = 25; *AtmSRIΔ^−/−^*: n = 8 and WT littermates: n = 31. **B**. Cumulative survival following a higher dose of 13Gy WBI is shown. *Rad21* ^+/−^: n = 9 and WT littermates: n = 7.

*Rad21^+/−^* mutant and WT littermates were exposed to a single dose of 8 or 13Gy WBI and Kaplan-Meier survival curve was determined over 30 days post IR. Following a single dose of 8Gy WBI, one death was recorded for WT mice. By contrast, a significant reduction in post-irradiation survival was observed in *Rad21^+/−^* mutant mice, beginning on day 17 post IR (Fig. 3A). To examine these data in the context of radiation sensitivity, we treated a cohort of *Atm*-defective mice with one or two alleles harboring a kinase domain inactivation mutation found in AT patients (*AtmSRIΔ*) [28]. As expected, the *AtmSRIΔ* homozygous mutant mice were remarkably hypersensitive to IR, such that no animals survived beyond day 8. However, we found that *AtmSRIΔ* heterozygous mutant mice were more resistant to WBI than *Rad21^+/−^* mice, with 90% surviving beyond 30 days. These data make a simple but very important point that *Rad21^+/−^* heterozygous mice are extraordinarily hypersensitive to IR, even from the perspective of the archetypical IR sensitivity of mutations in the *Atm* gene.

Exposure to a higher dose (13Gy) of WBI resulted in 100% mortality in both *Rad21^+/−^* and WT littermates by day 8 post-treatment (Fig. 3B). The death of *Rad21^+/−^* animals occurred as early as day 4 post-IR and ∼38% *Rad21^+/−^* mice died by day 6, when no mortality was recorded for WT mice. The reduced survival following WBI in *Rad21^+/−^* mice was presumed to be due to the consequences of greater tissue damage. This was then investigated in more detail.

### *Rad21^+/−^* mutant animals display GIT radiosensitivity

In adult mammals, the GIT represents one of the most sensitive tissues to IR [29]. Given that our immunostaining revealed that RAD21 protein is abundantly expressed in crypt epithelial cells of both small and large intestines (Fig. S5), we predicted that WBI may have greater effect on *Rad21^+/−^* mice compared to WT animals. We therefore performed a detailed characterization of GIT damage in *Rad21^+/−^* and *AtmSRIΔ* homozygous mutant mice after WBI treatment. IR-induced GI crypt cell damage can be quantitatively analysed in the small intestine (SI) using the well-established *in vivo* microcolony assay for crypt survival [30], [31]. In mouse SI, surviving crypts, defined as consisting of 10 or more cells with prominent nuclei and little cytoplasm, are readily distinguishable from non-surviving crypts between 3 to 4 days post-IR [30], [31].

**Figure 4:**
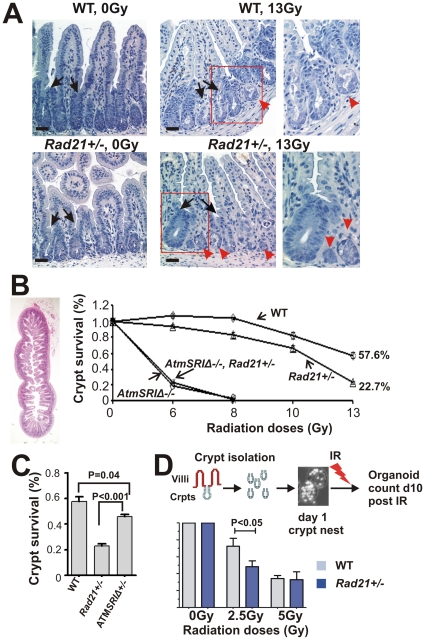
*In vivo* and *ex vivo* small intestinal crypt survival following radiation exposure. **A**. Hematoxylin-stained transverse sections of small intestines. Representative images of small intestinal crypts of unirradiated and irradiated animals. Irradiated samples shown were obtained at 3.5 days post 13Gy WBI. Non-surviving crypts are indicated by red arrowheads and surviving crypts by black arrows. Right panels: enlarged images from boxed areas. Scale bars = 20 µm. **B**. Crypt survival determined by the microcolony assay. Surviving crypts were counted 3.5 days post WBI at incremental doses as shown, in at least six whole circumferences of small intestine cross-sections (left panel) and expressed relative to unirradiated animals. The number of animals used for each data point is listed in Table S1. Error bars = SEM. **C**. Comparison of crypt survival in *Rad21* and *Atm* mutant animals 3.5 days post 13Gy WBI. WT and *Rad21^+/−^* data shown areas in B. The number of animals used is as follows: *Rad21^+/−^*: n = 7; AtmSRIΔ^+/*−*^ *:* n = 5; WT: n = 7. Error bars = SEM. **D**. *Ex vivo* crypt organoid survival. Diagrammatic presentation of the assay is shown above the graph. Day 1 organoid nuclei were visualised with Hoechst 33258 to show live cells. Data represent the percentage of organoids formed 10 days post IR at the doses indicated relative to unirradiated controls. The number of animals used is as follows: WT: n = 5; *Rad21^+/−^*: n = 7; four replicates were counted for each animal. Error bars = SEM.

We first determined the consequences of WBI on crypts at day 3.5, a time when we and others have observed maximum post-IR crypt damage, and when recovery is initiated. Following WBI, some crypts shrunk as a result of extensive cell depletion and appeared as ‘ghost’ structures (Fig. 4A). Taking a more quantitative approach, we assessed the percentage of SI crypt survival at 3.5 days post WBI in *Rad21^+/−^* and WT littermates following graded doses of 0, 6, 8, 10 and 13Gy, using the microcolony analysis. There was no statistical difference in the number of crypts between the different genotypes under homeostasis and without IR (Table S1). *Rad21^+/−^* mice exhibited a dose-dependent decrease in crypt survival that is greater than WT (Fig. 4B). The difference between the genotypes was highest at 13Gy, with more than two-fold higher surviving crypts recorded in WT (57.6%) compared to *Rad21^+/−^* (22.7%) mice (Fig. 4B and Table S1). Interestingly, a slight increase in crypt number in WT mice following WBI at 8Gy was observed (Fig. 4B and Table S1). Presumably, this increase in the number of crypts is associated with IR-induced crypt regeneration. These data clearly show that *Rad21^+/−^* mice were more susceptible to IR-induced SI damage compared to WT.

When *AtmSRIΔ* homozygous mutant SI were examined, it was apparent that they were substantially more IR sensitive than WT mice (Fig. 4B). To test for synthetic interactions between *Atm* and *Rad21* in the *in vivo* response of the SI to IR, we intercrossed these mice. When challenged with WBI, the high degree of SI sensitivity of the *AtmSRIΔ* homozygous mutant SI was not enhanced in the presence of our *Rad21* mutant allele. These data further supported the notion that, in mammals, the function of *Rad21* contributes to the same pathway as *Atm*. The SI of mice with a single *Rad21* mutant allele was demonstrably, reproducibly and statistically more IR sensitive than the *AtmSRIΔ* heterozygous SI (Fig. 4C).

To assess whether the reduced crypt survival in *Rad21^+/−^* mice is the result of intrinsic epithelial or secondary damage (for example, as a result of endothelial cell damage), we developed an *ex vivo* crypt survival assay using organoids established from isolated SI crypts. In this assay, crypts were irradiated *ex vivo* and the organoids which formed were counted 10 days post irradiation. At an IR dose of 2.5Gy, 72% of organoids were formed from WT crypts. Only 48% organoids were formed from irradiated *Rad21^+/−^* crypts, significantly lower than that of WT (Fig. 4D). We noted that no significant difference was observed between WT and *Rad21^+/−^* crypts at a higher IR dose of 5Gy. We posit that this higher dose is above a threshold level even for WT SI, for extensive crypt death in both genotypes. Nevertheless, these data provide strong evidence that the crypt death in *Rad21^+/−^* mice is unlikely to be a consequence of toxicity secondary to endothelial dysfunction.

### Small intestinal epithelial cells of *Rad21^+/−^* mice are deficient in DSB repair and fail to resume crypt regeneration following IR

**Figure 5:**
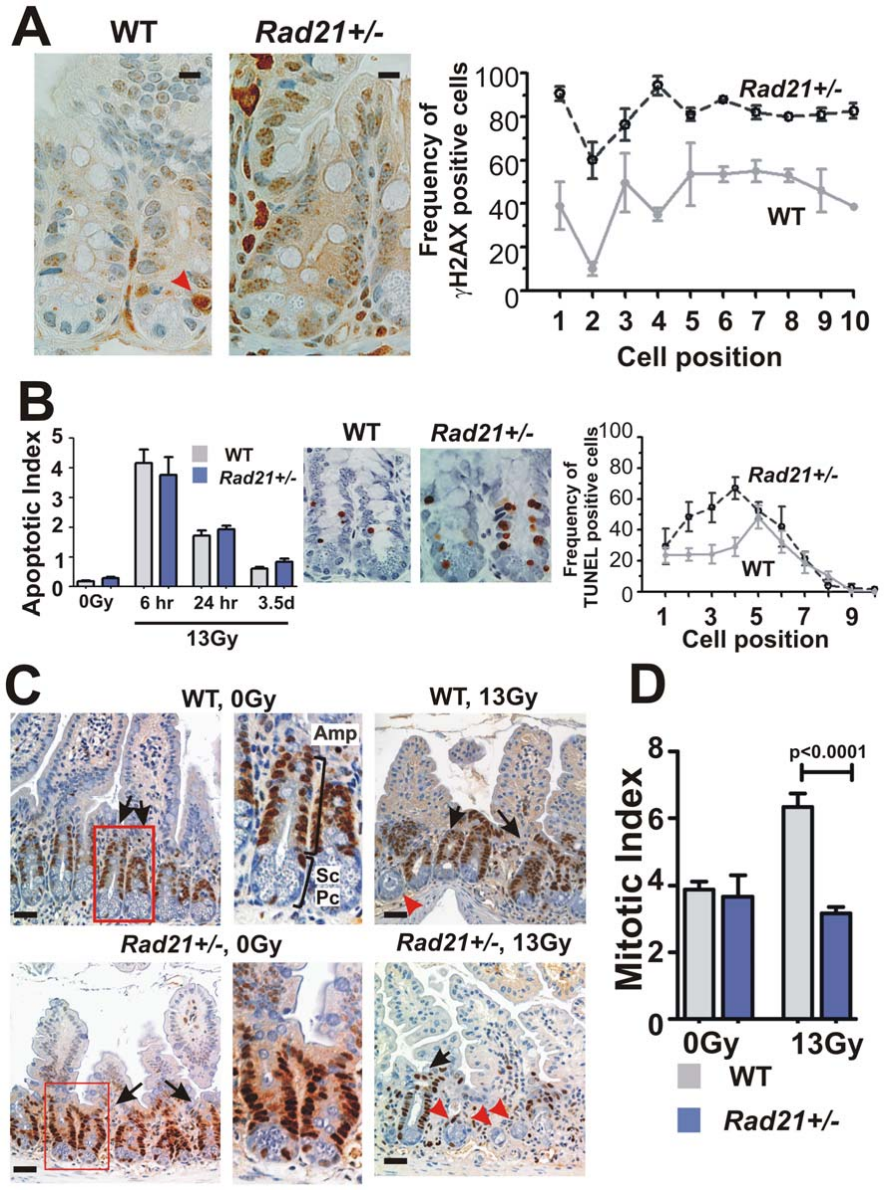
Cellular properties of small intestinal crypts post 13 Gy WBI. **A**. *γ*H2AX immunostaining of small intestinal crypt 6 hours after IR. Representative images of WT (left panel) and *Rad21^+/−^* (middle panel) are shown. The right panel shows the frequency of *γ*H2AX-positive cells scored according to the cell positions relative to the base of crypts. At least 20 crypts per animal and three animals per genotype were scored. Red arrowhead: apoptotic cells. Scale bars = 20 µm. **B**. Apoptotic cells determined by TUNEL staining at indicated time points post IR. Middle panels show the representative images of TUNEL-positive cells in crypts 6 hours post IR. Right panel show the frequency of TUNEL-positive cells, scored according to cell position. At least 20 crypts per animal and three animals per genotype were scored to determine the frequency. **C**. PCNA immunostaining of crypts 3.5 days post IR. Middle panels: enlarged images of boxed areas showing three compartments of a crypt: Paneth cells (Pc), putative stem cells (Sc) and transient amplifying cells (Amp). Black arrows: surviving crypts; Red arrowheads: non-surviving crypts. Scale bars = 20 µm. **D**. Mitotic indices determined by phospho-Histone H3 immunostaining at 3.5 days post IR. At least four whole circumferences of small intestinal cross-sections were scored per animal. The number of animals scored is as follows: WT 0Gy n = 5, 13Gy n = 6; *Rad21* ^+/−^ 0Gy n = 3, 13Gy n = 10. Error bars = SEM.

Given that *Rad21^+/−^* MEFs exhibited impaired DSB repair, we hypothesised that enhanced radiation sensitivity of *Rad21^+/−^* crypt cells was associated with their inability to repair IR-induced DSBs. We therefore examined the presence of γH2AX foci in the small intestinal crypts of *Rad21^+/−^* and WT mice 6 hours post WBI. Distinct γH2AX foci were detected in the crypts of both *Rad21^+/−^* and WT mice, indicating the presence of DSBs (Fig. 5A). To characterise DNA repair according to cell type, γH2AX-positive cells were scored on a positional basis from the base to the mid-region of crypts, *i.e.* position 1 to position 10, which correspond to the combined Paneth cell, stem cell and transient amplifying compartments [32], [33]. *Rad21^+/−^* mice showed significantly higher frequencies of crypt epithelial cells with γH2AX foci at all positions examined, when compared with WT (p<0.0001) (Fig. 5A). The persistence of γH2AX foci in high proportions of *Rad21^+/−^* crypt epithelial cells strongly suggests a deficiency in DSB repair and is consistent with the defect we showed in *Rad21* mutant cells.

Radiation induced cell death is thought to be a major factor contributing to subsequent crypt loss [34], [35]. We next examined the effect of WBI on crypt cell apoptosis. TUNEL staining of the SI at 6 hours, 24 hours and day 3.5 post WBI revealed that IR induced a significant increase in crypt cell apoptosis in both *Rad21^+/−^* and WT mice (Fig. 5B, left panel). The number of apoptotic cells per crypt (Apoptotic Index) was highest at 6 hours post IR and decreased sharply at 24 hours (Fig. 5B, left panel). By day 3.5, few apoptotic cells per crypt were observed. The apoptotic index was not significantly different between *Rad21^+/−^* and WT mice; although there is a moderately higher apoptotic index in *Rad21^+/−^* mice compared to WT at 24 hours and at day 3.5 post IR (Fig. 5B, left panel). We further determined the frequency of TUNEL-positive cells according to the cell position in crypts at 6 hours post WBI when the peak of apoptosis was observed. TUNEL-positive cells in *Rad21^+/−^* mice were found to be more frequent in the crypt base at cell positions 1 to 4 where cryptal basal cells (stem/progenitor-cells) and Paneth cells reside [35], [36] (Fig. 5B, right panel), suggesting that stem/progenitor cells in *Rad21^+/−^* are more susceptible to IR-induced cell death.

The increased frequencies of γH2AX- and TUNEL-positive cells at the crypt base raise the possibility that the stem cell compartment in *Rad21^+/−^* intestinal crypts may be more susceptible to IR damage and cell death. The recruitment of stem cells to a proliferative state has been shown to be critical for crypt regeneration and GI recovery following IR-induced damage. We therefore assessed crypt cell proliferation in the SI of *Rad21^+/−^* and WT mice at 3.5 day post IR, a time when crypt regeneration occurs, using PCNA as a surrogate marker of proliferation. In unirradiated mice of both genotypes, “strings” of PCNA-positive cells were detected in the mid-crypt region, corresponding to the transient amplifying cells (Fig. 5C). At 3.5 days post 13Gy WBI, PCNA-positive cells were present in the majority of crypts in WT mice, indicative of active cell proliferation associated with crypt regeneration (Fig. 5C). In irradiated *Rad21^+/−^* mice, arrays of PCNA-positive cells were detected in “surviving” crypts but in the majority of crypts only a few scattering PCNA-positive cells were observed (Fig. 5C). We further examined proliferation using a mitotic-specific protein marker, phospho-histone H3 (PH3) in SI crypts to ensure that the PCNA staining was not being influenced in the WT crypts by PCNA-associated DNA repair functions [37].

Consistent with our PCNA staining, abundant PH3-positive cells were detected in surviving crypts of both *Rad21^+/−^* and WT mice (Fig. 5D). However, the number of PH3-positive cells per crypt (Mitotic Index) increased nearly two fold in irradiated WT mice compared to unirradiated animals, suggesting active mitoses associated with crypt regeneration (Fig. 5D). In irradiated *Rad21^+/−^* mice, the mitotic index remained at the similar level as unirradiated animals, but was significantly lower (p<0.0001) compared to irradiated WT mice (Fig. 5D). Taken together, these results strongly suggest that defective DSB repair and increased cell death within the region of the stem/progenitor cell compartment impairs crypt regeneration, leading to enhanced SI radiosensitivity in *Rad21^+/−^* mice.

### Colonic crypt epithelial cells of *Rad21^+/−^* mice fail to undergo mitotic arrest

**Figure 6:**
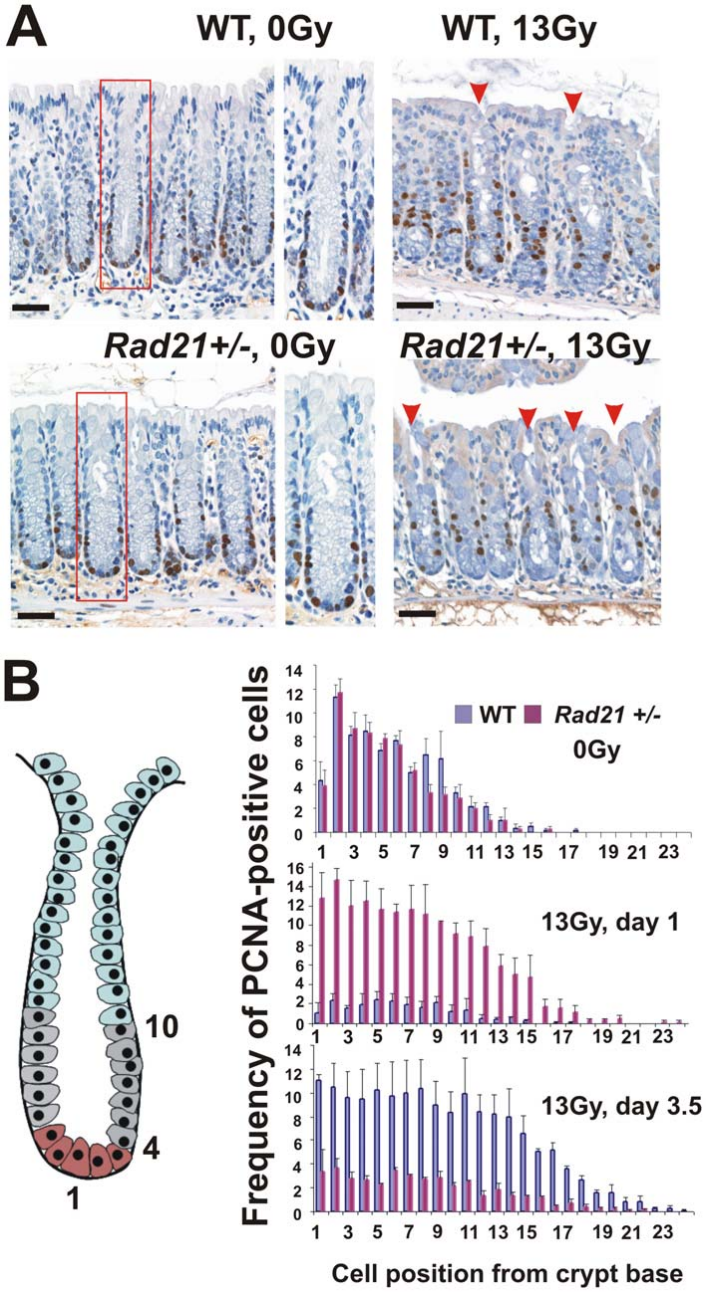
Kinetics of crypt epithelial cell proliferation in the large intestine post 13Gy WBI. **A**. Representative images of PCNA immunostained crypts at 3.5 days post IR. Middle panels: enlarged images of boxed areas. Red arrowheads indicate full-length crypts. Scale bars = 20 µm. **B**. Quantitative analysis of the frequency of PCNA-positive cells per 20 crypts at indicated time points post IR. Cell positions shown are relative to the bottom of crypts. The right panel shows a diagram of a typical colonic crypt. Three epithelial compartments shown are: stem/progenitor cells (brown); transit amplifying cells (grey); differentiated cells (light blue). Three animals were scored per data point. Error bars = SEM.

We further performed a detailed examination of the response of colonic crypt cells to IR, as radiation damage to the large intestine (colon) is similar to that of the SI but occurs at a slower rate. Colon damage doesn't form a prominent component of WBI syndromes, as its functional consequences are not as marked as for the SI. Nevertheless, in addition to its more regular crypt morphology as compared to the SI, it provides another opportunity to characterise the kinetics of crypt regeneration. We scored the frequency of PCNA-positive cells by colonic crypt cell position at day 1 and 3.5 post IR, to assess whether proliferation occurred in colonic crypt epithelial cells (Fig. 6A, 6B). The cell located in the centre of crypt base was designated as cell position 1 and cells that stained for PCNA were accordingly assigned to cell positions 1 or more up the crypt wall. In this fashion, data generated from the sides of both crypt walls can be pooled [38]. In unirradiated *Rad21^+/−^* and WT mice, the highest frequencies (8 to 12%) of PCNA-positive cells were detected at the positions 2 to 10; where the stem cells and lower transient amplifying population exist (Fig. 6B), with no significant difference between *Rad21^+/−^* and WT mice (Fig. 6B). At day 1 post IR, the frequency of PCNA-positive cells was dramatically reduced to 2–3% at these cell positions in irradiated WT colonic crypts (Fig. 6B). By contrast, the frequency of PCNA-positive cells at these positions in *Rad21^+/−^* mice remained similar to, or slightly increased following IR (Fig. 6B). Strikingly, the frequencies of PCNA-positive cells in irradiated *Rad21^+/−^* animals were significantly higher at all cell positions compared to that of irradiated WT (Fig. 6B). This response in *Rad21^+/−^* mice was unexpected and indicated that characteristic G1/S and G2/M checkpoints normally invoked by IR [39] were not being activated in the mutant GIT. Additionally, the frequencies of PCNA-positive cells at the positions 11 to 15 were noticeably increased following IR in the *Rad21^+/−^* crypts (Fig. 6B), consistent with an expected IR-induced recruitment of G0 cells into a proliferative state. At day 3.5 post IR, a striking reversed pattern was observed in WT animals, with a significant increase in the frequencies of PCNA-positive cells at cell positions 1 to 19. This was in sharp contrast to a significant reduction in the number of PCNA-positive cells at the majority of crypt cell positions in *Rad21^+/−^* mice (Fig. 6B). Together, these data suggest that *Rad21^+/−^* colonic crypt cells failed to initiate the canonical early IR-damage response of mitotic arrest. This severely compromised the subsequent process of crypt regeneration in mutant *Rad21^+/−^* mice.

### *Rad21* deficiency leads to an enhanced IR sensitivity in bone marrow stem cell compartments

Bone marrow contains stem cells, progenitor cells and their progeny, that are exquisitely radiosensitive [40]. Bone marrow suppression is the most recognized form of IR-induced damage after WBI in mammals, leading to irreversible depletion of the bone marrow stem cell reserve. To characterise IR-induced bone marrow damage in *Rad21^+/−^* animals, we investigated the hematopoietic response in *Rad21^+/−^* mice.

**Figure 7:**
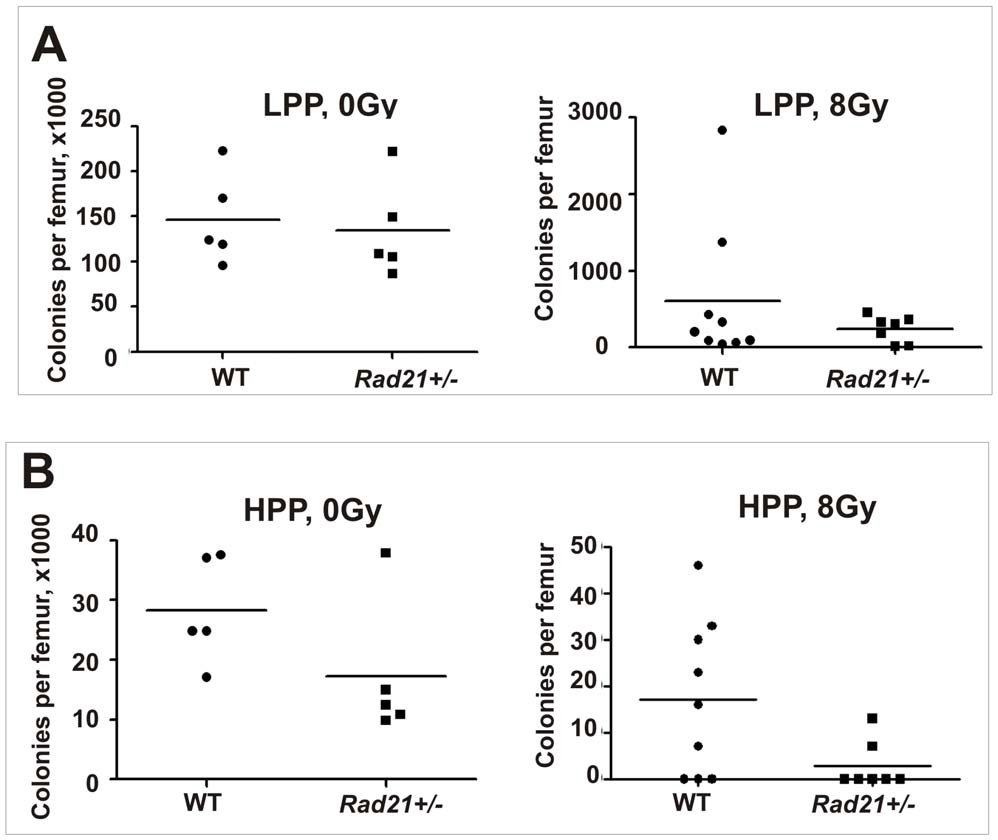
Basal and post-irradiation cloning efficiencies of bone marrow low proliferative potential-colony forming cells (LPP-CFC) and high proliferative potential-colony forming cells (HPP-CFC). Irradiated animals were assayed 10 days post 8Gy WBI. Data generated from two independent experiments and total animals used: WT 0Gy n = 5; 8Gy n = 10; *Rad21^+/−^* 0Gy n = 5, 8Gy n = 7. Horizontal bars represent mean. **A**. LPP -CFC. **B**. HPP-CFC.

We used a surrogate assay to measure stem-like cells by counting high proliferative potential-colony forming cells (HPP-CFC) in the hematopoietic stem cell compartment and the low proliferative potential-colony forming cells (LPP-CFC), characteristic of the committed progenitor cell pool [41]. Bone marrow cells from femurs of mutant and WT littermates were analysed at day 10 post 8Gy WBI. The result showed that LPP-CFCs were significantly reduced following IR, with approximately 0.1% cell survival. Although the average number of LPP-CFC in *Rad21^+/−^* mice appeared to be lower compared to WT, this was not significant so (p>0.05) (Fig. 7A). We noted that WT mice displayed greater individual variation in cloning efficacy than *Rad21^+/−^* mice. Similarly, the number of HPP-CFC in *Rad21^+/−^* mice was slightly but not significantly reduced compared to WT following IR (p = 0.205) (Fig. 7B). These data suggest impaired post-irradiation bone marrow stem cell clonogenic regeneration in *Rad21^+/−^* mice. It remains to be determined whether the bone marrow stem cells and progenitor cells recover to normal levels, or remain suppressed in *Rad21* mutant mice. Successive transplantation models might be instructive in probing this possibility.

## Discussion

### Identification of *Rad21* cohesin as a novel mammalian radiation responsive gene

Pioneering studies established *Ataxia telangiectasia* patients as a most radiation sensitive group of patients [1]. Being an autosomal recessive disorder, individuals with two *Atm* mutant alleles manifest a broad spectrum of neurological, oncological and immunological pathologies but fortunately the population frequency of *Atm* patients is relatively low. However, *Atm* carriers are far more frequent (around 1∶200) and it has been an ongoing concern that some of these individuals are likely to be IR sensitive even though this has been difficult to demonstrate [42]. In contrast, for a different endpoint, female *Atm* carriers have a clearly elevated risk for developing breast cancer [5]–[7]. Mouse models of *Atm* deficiency largely recapitulate the human AT phenotypes [28], [43]. Hence, some pathologies in *Atm* patients and mouse models, are evident without overt IR exposure but unlike *Rad21* homozygous mutant mice, they are viable. As *Rad21* is an essential gene and *Rad21^−/−^* embryos die very early in development, we investigated mice with only one mutant allele. This proved very instructive. The observations made here with *Rad21* ^+/*−*^ mice indicate that one *Rad21* allelic mutation confers a level of IR sensitivity that eclipses that observed in *Atm* heterozygous mice and accordingly raises the issue that should cancer patients with *Rad21* or potentially other cohesin mutations be subjected to RT they may suffer adversely from standard RT protocols.

WBI studies initially highlighted a reduced animal survival in *Rad21^+/−^* mice. Furthermore, the absence of genetic interaction with *Atm* mutant mice is consistent with cohesin functioning downstream of Atm signalling [15], [23], [44]. More focused studies that explored tissues that impact tolerance doses of RT, such as the GIT and bone marrow, revealed that these tissues were indeed hypersensitive to IR. It was most apparent that *Rad21^+/−^* mice are hypersensitive to the toxic effects of RT when compared to wild type littermates and this was manifested by crypt losses in both SI and colon. Importantly, the kinetics of the responses were different in these two tissue compartments with the notable observation that in the colon of *Rad21^+/−^* mice the characteristic and early (within the first 24 hours) shut down of proliferation following IR was absent, which would allow cells to progress with the burden of potentially lethal DNA damage. Our *in vivo* characterisation of *Rad21* ^+/*−*^ mutants was thus reminiscent of a classical feature of *Atm*-mutant cells, that of checkpoint failure, including radioresistant DNA synthesis [45]. Collectively, these novel data indicate that *Rad21* plays a fundamental role(s) in the IR response in whole mammals. *Rad21^+/−^* mutant animals are thus a unique resource for dissecting the molecular determinants of IR responses in the context of whole mammals.

### Mouse *Rad21* cohesin is required for HR

*Rad21* was implicated in promoting homologous recombination in fission yeast [8], [13]. Although the role of yeast RAD21 cohesin in mediating chromosome segregation was found to be conserved in mammals, there has been no definitive data to support a role for mammalian RAD21 in HR. We found that a reduction of *Rad21* gene dosage resulted in a significant reduction in the efficiency of gene targeting in mouse ES cells and in the frequency of MMC-induced SCEs in MEF cells, both processes mediated by HR [21], [46]. Our finding provides the strong evidence that RAD21, and/or the cohesin complex, directly or indirectly promotes HR in mammalian cells. Consistent with this finding, we showed that *Rad21^+/−^* ES cells were exquisitely sensitive to MMC. The repair of MMC-induced DNA lesions involves multiple DNA repair pathways, with HR and other HR-dependent DNA repair pathways, i.e. Fanconi anaemia (FA) and nucleotide-excision repair (NER), being the main repair pathways [19]. The observed MMC sensitivity in *Rad21^+/−^* ES cells supports a role for RAD21 in HR. Furthermore, this result suggests that RAD21 may be involved in one or more these DNA repair pathways.

We did not observe a significant reduction in the survival of *Rad21^+/−^* ES and MEF cells compared to that of WT cells following IR treatment. However, we found that IR resulted in significantly higher level of γH2AX foci in *Rad21^+/−^* MEF cells when compared with WT cells, suggesting impaired DSB repair in *Rad21^+/−^* MEF. Further, *Rad21^+/−^* MEFs have an elevated level of NPB and MN following IR. NPB and MN have been shown to originate from abnormal chromosomal structures such as dicentric chromatids, ring chromatids and acentric fragments [47]. These abnormal chromosomal structures form when cells undergo DNA replication in the presence of unrepaired DSBs and repair after replication, resulting in misjoined broken chromosome ends [47]. It is therefore likely that higher levels of NPB and MN observed in *Rad21^+/−^* cells are a consequence of the impaired DSB repair in combination with failure in cell cycle arrest following IR. Consistent with this proposition, our results showed that a significantly higher percentage of *Rad21^+/−^* MEF cells continued to undergo DNA replication and mitosis following IR.

The absence of significant IR sensitivity in *Rad21^+/−^* cells is in contrast to the marked IR-sensitivity observed in *Rad21* yeast mutants. The differences in DSB repair mechanisms between yeast and mammalian cells may explain, at least in part, the modest IR sensitivity in *Rad21^+/−^* cells. Unlike yeast which predominately uses a high-fidelity HR to repair DSBs [48], mammalian cells employ two distinct mechanisms for the resolution of DNA DSBs: the main pathway, non-homologous end-joining (NHEJ), and the other, the HR pathway [49]. HR is primarily used during S/G2 phase of cell cycle when sister chromatids are available as templates for repair. Although *Rad21^+/−^* cells are deficient in HR repair, it is possible that these cells use the NHEJ pathway to repair IR-induced DSBs, which in turn alleviates the IR sensitivity of *Rad21^+/−^* cells. Consistent with this proposition, a most recent study showed that radiation-induced DSB repair was impaired in G2, but not G1 phase in *Rad21*-depleted cells [50]. Further analysis using compound mutant mice models with either HR or NHEJ deficiency may assist in elucidating the role of cohesins in this context.

### Failure of crypt regeneration underpins GI sensitivity in *Rad21^+/−^* animals

Radiation resulted in a higher percentage of the crypt death in the SI in *Rad21* ^+/*−*^ animals compared to WT. IR damage to the GIT in mice is thought to result from two main scenarios: (i) as a direct consequence of damage to epithelial crypt cells [51] or, a more recent and hotly debated suggestion (ii) as a result of damage secondary, for example, to endothelial cell apoptosis [52]. We consider the possibility of secondary damage unlikely, as no post-IR microscopic evidence of endothelial apoptosis was seen in mutant intestines. To more definitively examine which of these mechanisms may apply to *Rad21* mutant cells, we performed an *ex vivo* crypt organoid assay; no angiogenesis occurs within these bodies. The data clearly showed that *Rad21* ^+/*−*^ crypt organoids are also more sensitive to IR than WT, without the possibility of endothelial-damage–generated consequences. Hence, the observed GI sensitivity in *Rad21* ^+/*−*^ mice is likely to be the result of direct damage to crypt epithelial cells.

Following radiation, a temporary mitotic arrest and subsequent cell death occurs [39]. These events comprise some of the key early responses of the GIT to radiation-induced damage. Cell death and lack of cell proliferation in combination with continued programmed cell shedding at the apices of villi results in extensive depletion of crypt cells, leading to crypt shrinkage and perturbed morphology [35]. Depending on the extent of damage, some shrinking crypts may resume proliferation within 24 hours to 36 hours post IR and those crypts may survive as a result of crypt regeneration, whereas others will ultimately die [31]. Our analyses revealed significantly less proliferative cells in the SI crypts of *Rad21* ^+/*−*^ mice 3.5 days post IR, suggesting that the process of crypt regeneration is severely compromised. We found that TUNEL-positive cells occurred more frequently at the crypt bases 6 hours post IR in *Rad21* ^+/*−*^ mice than that of WT animals. The base of SI crypts contains stem cells which are recruited from quiescence to a proliferative state in response to IR damage. Therefore, the lack of crypt regeneration in *Rad21* ^+/*−*^ mice could be due to the enhanced killing of their crypt stem cells by IR, leading to regeneration insufficiency of surviving clones. A second possibility exists: that unlike WT cells, the mutant crypt cells fail to withdraw from the cell cycle to repair lethal damage. This view was formed based on our results showing that the frequency of PCNA-positive cells at day 1 post IR in the irradiated *Rad21* ^+/*−*^ animals remained similar to that of unirradiated mice, suggesting ongoing active cell proliferation and failure to initiate cell cycle arrest. This proposition was further supported by our finding that *Rad21* ^+/*−*^ MEFs are defective in cell cycle arrest following IR. Cells with defective cell cycle checkpoints have been shown to be more susceptible to death by mitotic catastrophe, after treatment with DNA-damaging agents [53]. Mitotic catastrophe, also known as reproductive death, occurs because cells enter mitosis in the presence of residual or misrepaired DNA DSBs, leading to the propagation of mutations and chromosomal aberrations which ultimately results in cell death [54]. Further, we found that there was a higher incidence of cells with radiation-induced γH2AX foci at the crypt bases in *Rad21* ^+/*−*^ mice. We noted a higher level of apoptosis in *Rad21* ^+/*−*^ animals at later time-points (day 1 and day 3.5 post IR). It is therefore plausible that mitotic catastrophe in crypt cells is consequential to their DSB-induced proliferative response during crypt regeneration in *Rad21* ^+/*−*^ animals. Taken together, our data suggest that the GI sensitivity of *Rad21* ^+/*−*^ animals may be attributed to the impairment of crypt regeneration as a result of enhanced killing of crypt stem cells in combination with a failure in mitotic arrest followed by mitotic catastrophe.

It is intriguing that the severe sensitivity of *Rad21* ^+/*−*^ animals is not reflected in *Rad21* ^+/*−*^ ES and MEF cells. Multiple factors, such as the difference between cellular- and tissue-specific responses, may have contributed to the discrepancy. For example, small intestinal radiosensitivity is known to be largely associated with the rapid turn-over of small intestinal crypt cells [29]. Unlike ES and MEF cells, the epithelium (crypts and villi) of small intestines consists of cells that are short-lived and extruded within 3–5 days as they migrate up to the tip of villi. The renewal of crypt epithelial cells largely depends on a sub-population of stem cell progenitors residing at the base of crypts. Our results showed that following IR, *Rad21^+/−^* crypt cells fail to undergo cell-cycle arrest and continue to migrate towards the tip of crypts where they will be shed. Furthermore, we showed that the stem cell progenitors of *Rad21^+/−^* crypts are more susceptible to IR-induced cell death. The combination of these two factors inevitably results in the accelerated loss of crypt cells, leading to the enhanced crypt death. Presumably, it is these unique features of small intestinal crypts that revealed the severe intestinal radiosensitivity of *Rad21^+/−^* animals.

### GI sensitivity of *Rad21^+/−^* mutant animals - relevance to human radiation responses

The systematic and mechanistic investigation of *Rad21*'s role in the radiosensitivity of different normal tissues will be important for understanding the radiation responses in both tumours and normal tissues. Malignant tumors typically have a high proliferative fraction and in some ways behave similarly to tissues with rapid cell turnover, such as the SI. Our finding that SI crypt cells and long-lived hematopoietic cells of *Rad21^+/−^* mutant animals are more susceptible to killing by radiation raises the possibility that targeted depletion of *Rad21* in tumors would be of therapeutic utility and that the level of differential depletion between normal adjacent tissue and tumor cells would not need to be absolute to achieve a clinically relevant differential tumor cell kill. Conversely, our study has another important clinical implication. Cancer patients who sustain greater than expected side-effects in the SI from RT, may harbour mutations in, or dysregulation of, *Rad21*.

Clinical RS and its unfortunate functional consequences for cancer patients also provide a rationale for a predictive assay for testing for *Rad21* status prior to RT, as a form of molecularly individualized medicine. Those with *Rad21* defects could be spared irradiation or their doses limited, while WT cases could be considered for RT dose intensification. Given the typically steep dose-response curve for tumour cures by RT, the latter could conceivably result in enhanced cancer cures. Furthermore, human populations are expected to harbour a generally greater likelihood of heterozygous mutations than their quadratically-less-likely homozygous counterparts. Our finding of a gene dosage effect for mammalian *Rad21* IR sensitivity thus suggests that if *Rad21* function is conserved in mammals, *Rad21* DNA sequence variants, if present, should be discernable from background.

## Materials and Methods

### Targeted deletion of the *Rad21* gene and generation of mutant animals

Gene targeting and generation of mutant mice were carried out essentially as described previously [25]. Briefly, to construct the targeting sequence, 5′ and 3′ targeting arms of 1.5 kb and 5.0 kb, respectively, were generated by PCR from the W9.5 ES cell line [55]. The targeting construct was made by fusing a splice acceptor (SA) with an IRES *neo* gene cassette which has the SV40 polyA sequence to terminate the mRNA. The resulting targeting construct replaced the exon 2 of the *Rad21* gene, resulting in a null allele (Fig. S1A). Linearized DNA was introduced into W9.5 parental ES cells by electroporation and positive clones selected as described previously [25]. Protein expression in WT and three neomycin-resistant ES clones was examined by Western blot analysis. A ∼120 kDa Rad21 protein band was detected in each cell line (Fig. S1B). Judging from signal intensities, the clone 5 displayed a noticeably reduction of RAD21 protein level, while two other clones (83 and 96) had similar signal intensity as WT. The level of PCNA was similar in all cell lines, indicating that any the reduction of the RAD21 protein is not necessarily associated with changes in expression of this protein that is typically associated with cell proliferation. Targeting of this clone was confirmed by PCR and Southern blot analysis of genomic DNA. DNA Sequence analysis of PCR products confirmed the insertion of the *neo* gene into the correct site of the *Rad21* gene. Accordingly, ES clone 5 was used for microinjection into C57Bl/6J blastocysts. Chimeric offspring were obtained. Heterozygous *Rad21^+/−^* mice were generated by breeding. Tail DNA was used for PCR-based genotyping.

### Cell culture, ES cell targeting efficiency and mouse embryonic fibroblast cell lines

For the targeting efficiency assay, ES cells were cultured in BRL-conditioned medium as described elsewhere [46]. WT and the *Rad21^+/−^* ES cell lines were transfected with a Rad54-GFP knockin construct [20]. One week after selection with Puromycin, single-cell suspensions of surviving colonies were made following trypsinization and analysis by FACS on a green fluorescence (eGFP) versus forward scatter (FSC-H) plot. Results were plotted in a fluorescence (GFP) histogram as described previously [20]. Targeting efficiencies were determined using Modfit software (Beckton Dickinson) and are indicated in each histogram. *Rad54^−/−^* ES cell lines were used as gene targeting-defective controls.

MEF cell lines were established from E13.5 embryos as described [55]. The procedure was approved by the Peter MacCallum Cancer Centre Animal Experimental Ethics Committee (approval #1275 and #1346). Cells are maintained in DMEM supplemented with 10% FCS, 1% Penicillin and Streptomycin, and 0.2% 0.1 M β-mercaptoethanol.

### Clonogenic survival assays

WT and isogenic *Rad21^+/−^* ES cells were seeded in 35 mm plates at appropriate densities in duplicate plates following a single dose of graded γ-ray irradiation (0, 2, 4, 6 and 8Gy). Cells were cultured for 14 days and colonies containing more than 50 cells were scored as clonogenic survivors. For assaying MMC sensitivity, ES cells were seeded in 6-well plates at appropriate densities, allowed to adhere and treated with MMC for 3 hours. Following treatment, MMC was removed and plates washed. Cells were cultured in MMC-free medium for 14 days and surviving colonies of more than 50 cells were counted. Three independent experiments were performed for each assay.

### FACS analysis

MEFs were irradiated at 10Gy and harvested at 24 and 48 hours post IR. Unirradiated cells were used as controls. Cells were fixed in 70% ethanol and treated with propidium iodide and RNase A. DNA content was analysed by flow cytometry. For BrdU labelling, cells were incubated in 10 ìM BrdU for 45 min prior to harvesting. Cells were then fixed in 70% ethanol, washed and treated with 2 N HCl containing 0.5% TritonX-100 for 30 minutes. Following neutralisation with 0.1 M Na_2_B_4_O_7_, cells were incubated with a monoclonal anti-BrdU antibody (BD Biosciences) for 30 minutes. Alexa 488 anti-mouse IgG (Invitrogen) was used as the secondary antibody. Cells were stained with propidium iodide and analysed by flow cytometry. Cell cycle profiles were analysed using ModFit and FCS express 3 softwares.

### Chromosome spreads, SCE assay and Immunofluorescence

Metaphase chromosome spreads were prepared as described previously [56]. SCE was carried out essentially as described elsewhere [57]. Briefly, MEFs were cultured in the presence of 5 µM BrdU for 48 hours. Cells were then incubated in Colcemide (0.15 µg/ml final concentration) for 2 hours and chromosome spreads were prepared. For MMC-induced SCEs, cells were treated with MMC (6 µM final concentration) for 30 minutes prior to the addition of Colcemide. SCEs were scored in a minimum of 20 metaphase spreads with near-diploid chromosome content. Immunofluorescence was performed as described previously [56]. For γH2AX immumostaining, MEFs were irradiated at 10Gy and fixed 4 hours post IR. Unirradiated MEFs were used as basal controls. Primary antibodies were a rabbit polyclonal anti-γH2AX (gift from Dr W. Bonner) and a mouse monoclonal anti-α-tubulin antibodies (Sigma-Aldrich, used at 1∶1000 dilution). Secondary antibodies were Alexa-488 anti-mouse and Alexa-568 anti-rabbit antibodies (Invitrogen). Cells were counterstained with either DAPI or propidium iodide. Images were collected using an Olympus BX51 microscope and γH2AX foci were counted using Metaphorph software.

### Whole body irradiation

Animals of mixed (129/Sv X C57BL/6) background were housed in micro-isolators in a specific pathogen free (SPF) facility. Compound mutant mice of *Rad21^+/−^* and *AtmSRIΔ* [28] were generated by intercross. Adult mice (eight to ten week-old littermates) were exposed to a single dose of WBI (6Gy, 8Gy, 10Gy or 13Gy) using a Caesium-137 gamma source at the dose rate of 1.67 min/Gy. Following IR, mice were given water containing antibiotic (0.5 g/L Neomycin), except those used for bone marrow assays. Animals were checked daily for signs of stress, including abnormal posture (head down), ambulation (reluctance to move and unresponsive to activity), laboured breathing (panting or gasping), loss of appetite, rough coat and diarrhoea. Animals displaying signs of severe stress were culled. Animals used for tissue analysis sensitivities were sacrificed at specified time-points post WBI. For determining survival, animals were observed for 30 days. The procedure for animal experiments was approved by the Peter MaCallum Cancer Centre Animal Experimental Ethics Committee (approval #1275 and #1346).

### Histology

Mice were sacrificed by cervical dislocation following required time-points following WBI. Twelve segments of 0.5 cm in length were collected from the SI jejunum, fixed in 10% normal buffered formalin, embedded in paraffin and sectioned transversely. Because of the variation in radiation response in different regions of the colon, only the distal colon was used for analysis and sections were cut longitudinally [31], [51]. Sections were stained with hematoxylin & eosin (H&E).

### Microcolony assay

SI samples were collected at day 3.5 post WBI following various doses and processed asdescribed above. For each mouse, the number of surviving crypts was scored in at least six whole transverse SI sections according to the criteria set for microcolony assay [30], [31]. A surviving crypt was defined as containing 10 or more darkly stained nuclei, little cytoplasm and lying close together. Scoring was performed in a code-blinded fashion with samples identified only by histological block number. SI's from at least five mice were scored per data point.

### Immunohistochemistry (IHC)

IHC was performed using an Envision™+ kit (Dako) according to the manufacturer's instructions. Antigen retrieval was carried out in 10 mM Tris buffer and 1 mM EDTA pH 9.0 for 2 minutes in a pressure cooker (Biocare Decloker). Primary antibodies were a mouse monoclonal anti-PCNA antibody (Dako M0879 clone PC10, used at 1∶800 dilution), a rabbit polyclonal anti-Rad21 antibody (Abcam, used at 1∶200 dilution), rabbit polyclonal anti-γH2AX (gift from Dr W. Bonner) and rabbit polyclonal anti-phospho-histone H3 antibodies (Abcam, used at 1∶500 dilution). Slides were counter-stained with hematoxylin, dehydrated in ethanol and coverslipped.

Phospho-histone H3-positive cells were counted in the whole circumference of at least four SI cross-sections. MI was derived from the number of mitoses per crypt. PCNA-positive cells in large intestinal crypts were recorded on a positional basis using a central cell at the crypt base as position 1. Accordingly longitudinal crypt positions on both walls of the crypt can be assigned numbers 1, 2, 3 etc. and pooled for scoring purposes [38]. Only cells stained with strong intensity were scored. All other cells that stained either weakly or not at all were regarded as negative. At least 20 crypts per mouse were scored and a minimum of five mice per data point were assessed. Counting was done in a code-blinded fashion, with samples identified by the histology block numbers and genotypes only being revealed at the end of scoring.

### TUNEL staining

TUNEL staining, which detects DNA fragmentation in cells undergoing apoptosis, was done using the Apop-Tag Kit (Chemicon) following the manufacturer's instructions. Sections were counterstained with hematoxylin. Total numbers of TUNEL-positive cells in SI crypts were counted in the whole circumference of at least four cross-sections in a code-blinded manner. The apoptotic index describes the average number of apoptotic cells per crypt. The frequency of TUNEL-positive cells was determined by scoring at least 20 crypts per mouse and three animals per genotype.

### Intestinal organoids culture and *ex vivo* irradiation assay

Crypt nests obtained from small intestines were counted using trypan blue for viability stain and the required number of crypt nests was seeded in Matrigel (BD Biosciences) overlayed with 500 µl of DMEM/F12 (Sigma) containing 20 ng/ml EGF (BD Biosciences), 10 ng/ml bFGF (Roche), 500 ng/ml R-spondin (RnD Systems), 100 ng/ml Noggin (Peprotech) and B27 supplement (Invitrogen). Cultures were established on day 0 and irradiated the next day using a γ-cell irradiator. Viable organoids were scored 10 days post irradiation following incubation in MTT (3-(4,5-Dimethylthiazol-2-yl)-2,5-diphenyltetrazolium bromide).

### Colony forming cell (CFC) assay

Littermates were treated with a single dose of 8Gy WBI and sacrificed at day 10 post IR, along with unirradiated littermates. Bone marrow cells were harvested from one femur. Cells were plated in triplicates at 2,500 cells per 35 mm dish for unirradiated mice, or 1/10 and 1/100 per femur for irradiated mice using a double layer agar containing colony stimulating factor-1 (CSF-1) at 1×10^3^ U/dish), IL-3 at 25 U/dish, Interleukin 1alpha (IL-1alpha) at 800U/dish and stem cell factor (SCF) at 100 ng/dish [58]. Partially purified mouse uterus extract (PMEU) was used as a source of CSF-1. IL-3 was obtained from conditioned medium from a mouse mammary cell line. Plates were incubated in a 37°C incubator under 5% O_2_, 10% CO_2_ and 85% N_2_ for 14 days. Colonies (>50 cells/colony) were counted under an inverted microscope.

LPP-CFCs form, in the presence of CSF-1 alone, colonies containing 50 to 50,000 cells with a diameter of less than 0.5 mm. HPP-CFCs form, in the presence of multiple growth factors (i.e. CSF-1, IL-3, IL-1alpha and SCF), colonies of more than 100,000 cells with a diameter of greater than 0.5 mm. Colony number per mouse femur was determined. Colonies larger than 0.5 mm diameter were scored as HPP colonies and those less than 0.5 mm in diameter as LPP colonies.

### Western blot analysis

Protein extracts were prepared as described [56]. Western blots were probed with a polyclonal anti-Rad21 antibody (Abcam, used at 1∶500 dilution) and a polyclonal anti-SMC3 antibody (Abcam, used at 1∶1000 dilution). Signal was detected with chemiluminescence. The membrane was then stripped to remove any signal and re-probed with a monoclonal anti-PCNA antibody (DAKO) and a monoclonal anti-α-tubulin antibody (Sigma-Aldrich).

### Statistical analysis

Statistical analysis was performed using GraphPad Prism 5 software. Clonogenic survival post IR was analysed using the linear quadratic model and survival post MMC treatment was analysed using a two components exponential model. P values were determined using the Students T-test, one-way ANOVA test or Chi-square test. The Log-Rank test was used for cumulative survival analysis.

## Supporting Information

Figure S1 Targeted deletion of mouse Rad21 gene. A. Diagram of targeting construct. Exons are shown in solid bars. E: EcoRI; SA: slicing acceptor; IRES: internal ribosome entry site; NEO: neomycin. Insert: Southern blot analysis showing the confirmation of the targeted allele (asterisk). B. Western blot analysis of RAD21 protein level in ES cells. Two independent WT (Rad21+/+) cell lines and three Rad21+/− ES cell lines (#5, #83 and #96) were tested. The membrane was probed with anti-RAD21, and anti-SMC3 cohesin antibodies. Actin and PCNA antibodies were used as controls for loading and the proportion of S phase cells, respectively. Note: reduced RAD21 and SMC3 level in ES clone 5 compared to WT and the two other Rad21+/− clones.
(0.83 MB TIF)

Figure S2 Chromosomal and mitotic abnormalities of Rad21+/− MEFs. A. The frequency of cells with diploid and aneuploid chromosome content. Data represent the percentage of metaphase spreads prepared from passage 2 MEFs. There is a clear increase in the frequency of aneuploid cells in Rad21+/− MEFs compared to WT (p<0.05). The number of metaphase spreads scored was as follows: WT n = 61; Rad21+/− n = 113. B. Metaphase spreads showing the altered centric chromatin organization in Rad21+/− cells. Arrowheads: sister chromatids lying in parallel to each other with no apparent centric connection. Insert: enlarged images of the boxed region. The percentage of cells with this phenotype was clearly higher in Rad21+/− MEFs compared to WT (p<0.05). The number of mitotic cells scored: WT n = 41; Rad21+/− n = 34. C. Representative images of normal and abnormal mitoses. Chr.: chromosome. Arrows: chromosome bridges and lagging chromosomes. Arrowhead: a chromosome or chromosome fragment without apparent spindle attachment. The number of mitotic cells scored: WT n = 36; Rad21+/− n = 33.
(2.20 MB TIF)

Figure S3 Radiation induced-genomic instability. Nuclei of unirradiated and irradiated WT and Rad21+/− MEFs were visualised by DAPI staining. Abundant NPBs and MNs were detected in Rad21+/− MEFs following IR.
(2.52 MB TIF)

Figure S4 Immunostaining of IR-induced γH2AX foci in WT and Rad21+/− MEFs 4 hours post IR. DNA was counterstained with DAPI. Focus numbers in Rad21+/− cells are approximately 30 to 50 fold the number seen basally in these cells. Left panels: black and white micrographs showing γH2AX foci. Right panels: merged images of γH2AX foci (red) and DAPI (blue). Scale bars = 20 µm.
(1.85 MB TIF)

Figure S5 Immunostaining of RAD21 protein in mouse intestinal crypts. DNA was counterstained with DAPI. Focus numbers in Rad21+/− cells are approximately 30 to 50 fold the number seen basally in these cells. Left panels: black and white micrographs showing γH2AX foci. Right panels: merged images of γH2AX foci (red) and DAPI (blue). Scale bars = 20 µm.
(3.72 MB TIF)

Table S1 (0.10 MB DOC)
