# Correction: *Rad21*-Cohesin Haploinsufficiency Impedes DNA Repair and Enhances Gastrointestinal Radiosensitivity in Mice

**DOI:** 10.1371/annotation/12224797-353c-4e9c-92f3-a0de9b527415

**Published:** 2010-09-17

**Authors:** Huiling Xu, Kuhendra Balakrishnan, Jordane Malaterre, Matthew Beasley, Yuqian Yan, Jeroen Essers, Esther Appeldoorn, Jonathan M. Tomaszewski, Melisa Vazquez, Sandra Verschoor, Martin F. Lavin, Ivan Bertoncello, Robert G. Ramsay, Michael J. McKay

The 8th and 13th authors' names were spelled incorrectly. The correct name of the 8th author is Jonathan M. Tomaszewski. The correct name of the 13th author is Ivan Bertoncello.

