# Supplementary figures and images for: Rad21-Cohesin Haploinsufficiency Impedes DNA Repair and Enhances Gastrointestinal Radiosensitivity in Mice
Source: PLoS One. 2010 Aug 12;5(8):e12112. doi: 10.1371/journal.pone.0012112 (PMC2920816; doi:10.1371/journal.pone.0012112)

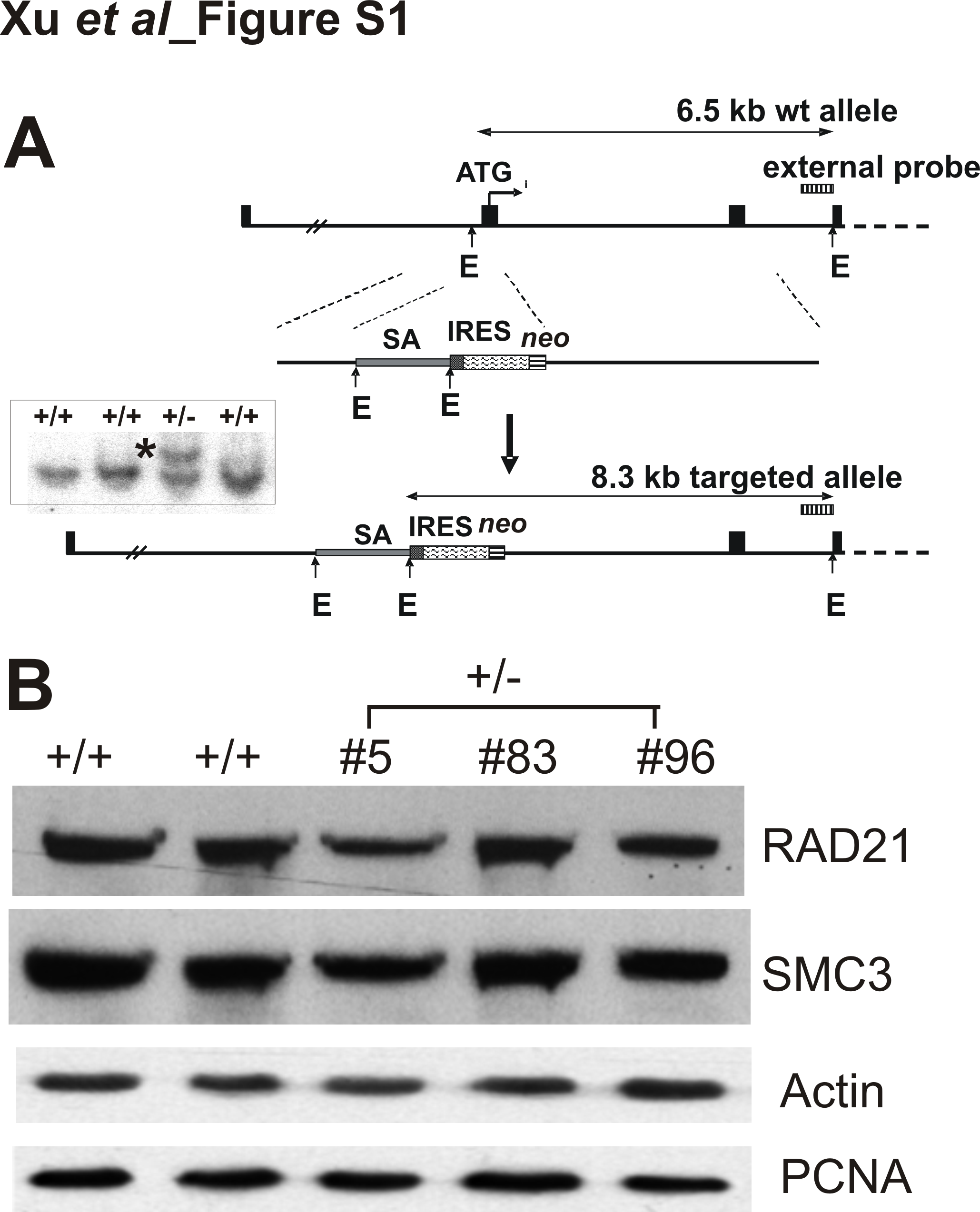

Supplement: Figure S1 — Targeted deletion of mouse Rad21 gene. A. Diagram of targeting construct. Exons are shown in solid bars. E: EcoRI; SA: slicing acceptor; IRES: internal ribosome entry site; NEO: neomycin. Insert: Southern blot analysis showing the confirmation of the targeted allele (asterisk). B. Western blot analysis of RAD21 protein level in ES cells. Two independent WT (Rad21+/+) cell lines and three Rad21+/− ES cell lines (#5, #83 and #96) were tested. The membrane was probed with anti-RAD21, and anti-SMC3 cohesin antibodies. Actin and PCNA antibodies were used as controls for loading and the proportion of S phase cells, respectively. Note: reduced RAD21 and SMC3 level in ES clone 5 compared to WT and the two other Rad21+/− clones. (0.83 MB TIF) [file pone.0012112.s001.tif]

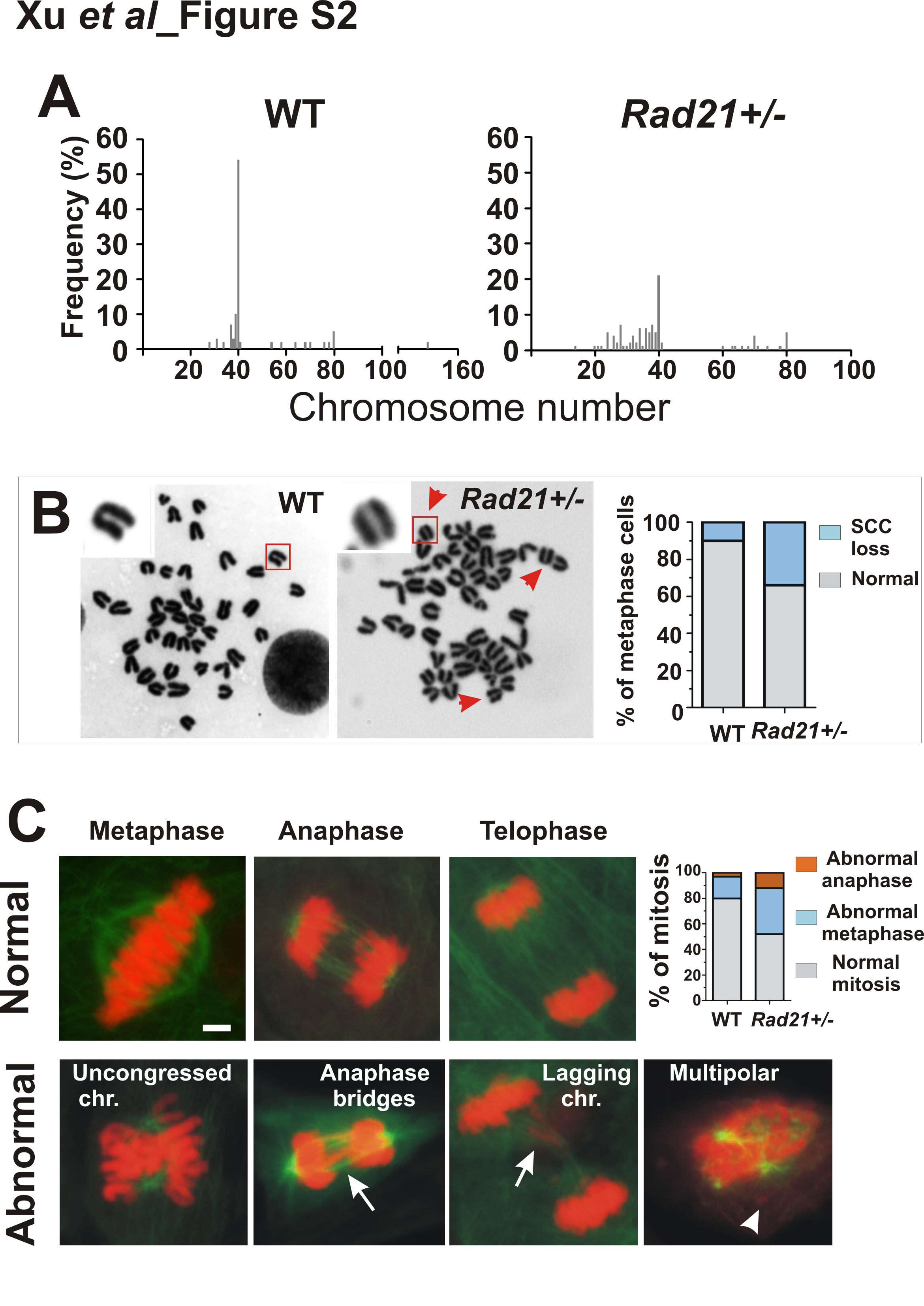

Supplement: Figure S2 — Chromosomal and mitotic abnormalities of Rad21+/− MEFs. A. The frequency of cells with diploid and aneuploid chromosome content. Data represent the percentage of metaphase spreads prepared from passage 2 MEFs. There is a clear increase in the frequency of aneuploid cells in Rad21+/− MEFs compared to WT (p<0.05). The number of metaphase spreads scored was as follows: WT n = 61; Rad21+/− n = 113. B. Metaphase spreads showing the altered centric chromatin organization in Rad21+/− cells. Arrowheads: sister chromatids lying in parallel to each other with no apparent centric connection. Insert: enlarged images of the boxed region. The percentage of cells with this phenotype was clearly higher in Rad21+/− MEFs compared to WT (p<0.05). The number of mitotic cells scored: WT n = 41; Rad21+/− n = 34. C. Representative images of normal and abnormal mitoses. Chr.: chromosome. Arrows: chromosome bridges and lagging chromosomes. Arrowhead: a chromosome or chromosome fragment without apparent spindle attachment. The number of mitotic cells scored: WT n = 36; Rad21+/− n = 33. (2.20 MB TIF) [file pone.0012112.s002.tif]

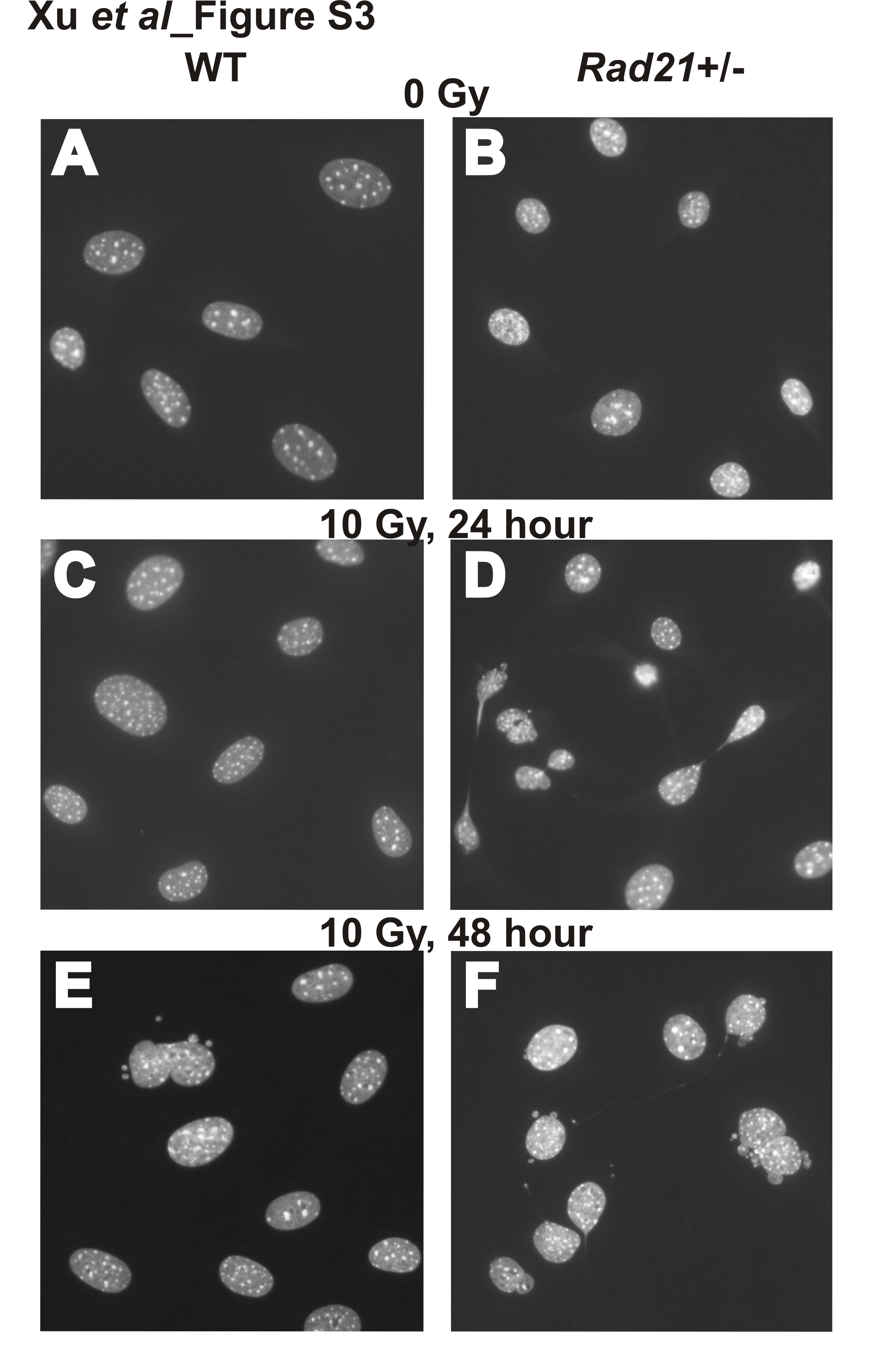

Supplement: Figure S3 — Radiation induced-genomic instability. Nuclei of unirradiated and irradiated WT and Rad21+/− MEFs were visualised by DAPI staining. Abundant NPBs and MNs were detected in Rad21+/− MEFs following IR. (2.52 MB TIF) [file pone.0012112.s003.tif]

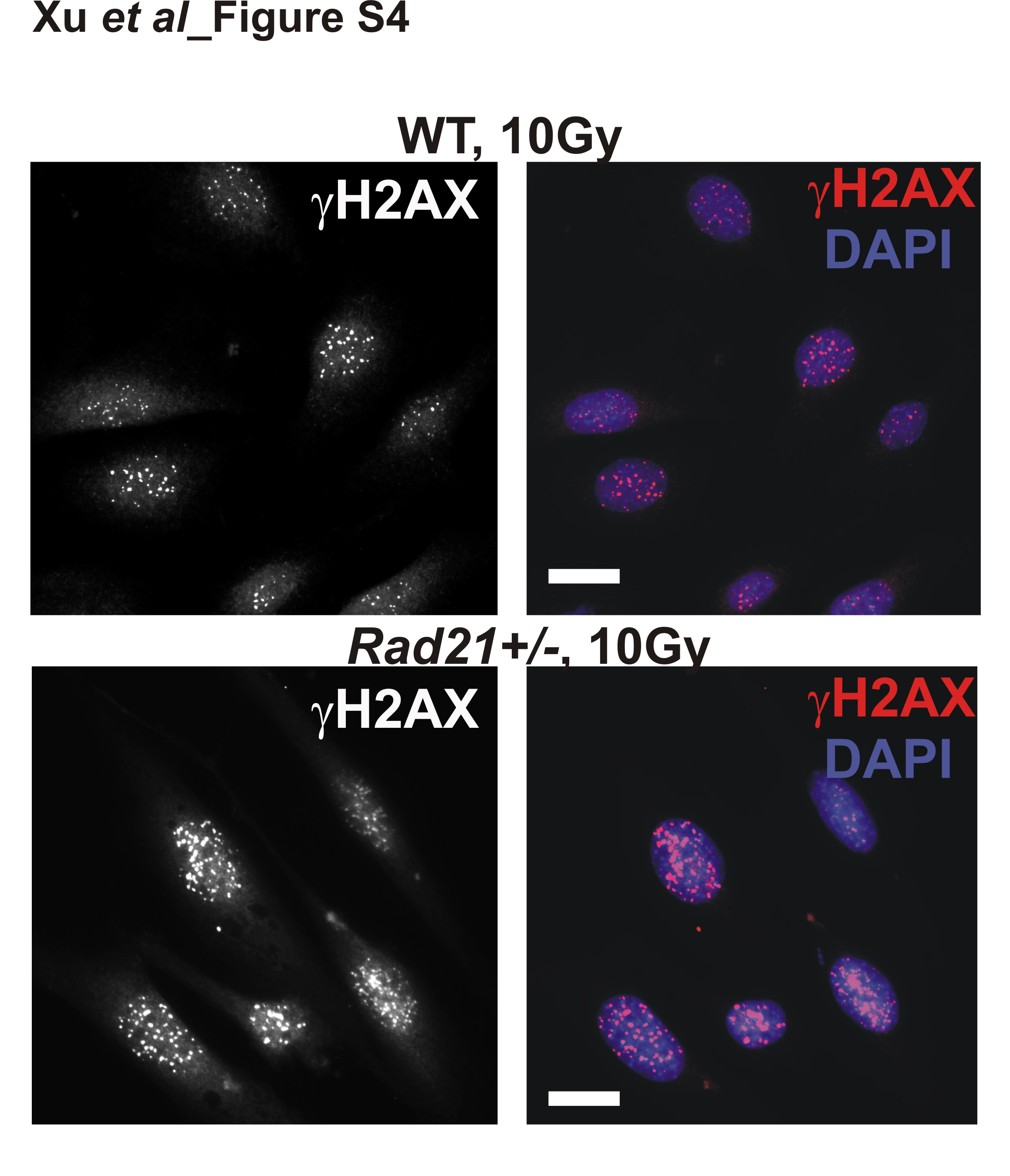

Supplement: Figure S4 — Immunostaining of IR-induced γH2AX foci in WT and Rad21+/− MEFs 4 hours post IR. DNA was counterstained with DAPI. Focus numbers in Rad21+/− cells are approximately 30 to 50 fold the number seen basally in these cells. Left panels: black and white micrographs showing γH2AX foci. Right panels: merged images of γH2AX foci (red) and DAPI (blue). Scale bars = 20 µm. (1.85 MB TIF) [file pone.0012112.s004.tif]

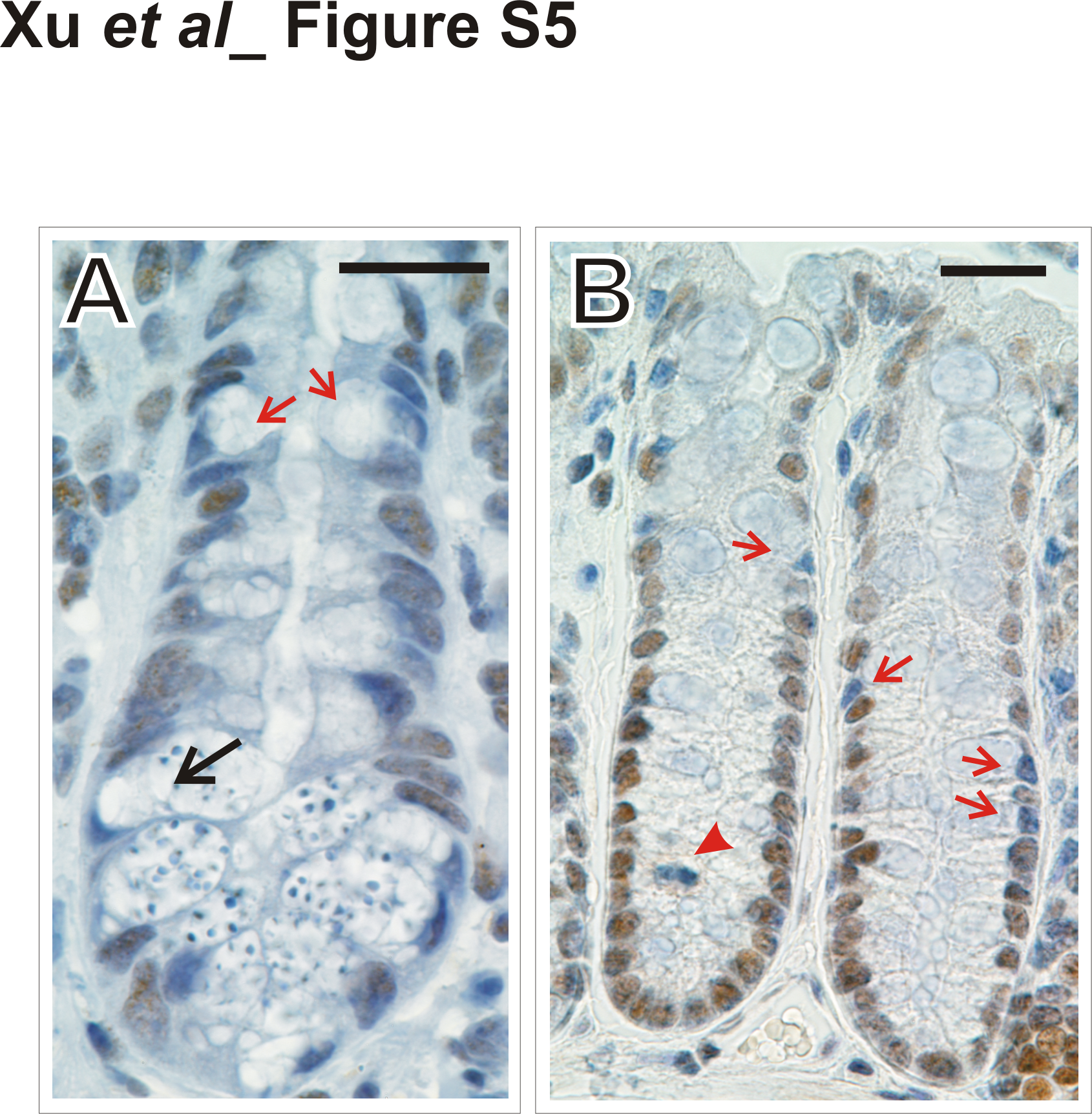

Supplement: Figure S5 — Immunostaining of RAD21 protein in mouse intestinal crypts. DNA was counterstained with DAPI. Focus numbers in Rad21+/− cells are approximately 30 to 50 fold the number seen basally in these cells. Left panels: black and white micrographs showing γH2AX foci. Right panels: merged images of γH2AX foci (red) and DAPI (blue). Scale bars = 20 µm. (3.72 MB TIF) [file pone.0012112.s005.tif]
