# Supplementary material for: Rad21-Cohesin Haploinsufficiency Impedes DNA Repair and Enhances Gastrointestinal Radiosensitivity in Mice
Source: PLoS One. 2010 Aug 12;5(8):e12112. doi: 10.1371/journal.pone.0012112 (PMC2920816; doi:10.1371/journal.pone.0012112)
Supplement: Table S1 — (0.10 MB DOC) [file pone.0012112.s006.doc]

**Table S1** Quantitative analysis of small intestine crypt survival determined by microcolony assay at day 3.5 following WBI.

|  | *WT* | | *Rad21^+/-^* | |  |
| --- | --- | --- | --- | --- | --- |
|  | *Crypt** | *% survival* | *Crypt** | *% survival* | *P value* |
| *Unirradiated* | *108.3 ± 2.8**(n= 5)* | *100 %* | *116.7 ±3.3**(n=6)* | *100%* | *0.091* |
| *6Gy* | *115.9 ±2.5**(n= 9)* | *107.6 %* | *97.3 ±1.6**(n=8)* | *83.4%* | *0.34* |
| *8Gy* | *112.5 ± 1.9**(n= 7)* | *103.9 %* | *97.3 ±3.6**(n=11)* | *83.4%* | *0.0064* |
| *10Gy* | *86.4 ± 2.9**(n= 7)* | *82.4 %* | *76.9 ±3.7**(n=9)* | *66.4%* | *0.0723* |
| *13Gy* | *57.6 ± 2.7**(n= 7)* | *53.3 %* | *26.8 ±1.7**(n=7)* | *22.7%* | *5.56E-07* |

* Surviving crypts ***±*** Standard Error of Mean (SEM)
